# Supplementary material for: Risk factors for decline in estimated glomerular filtration rate amongst Malawian adults living in rural Karonga: Protocol for a prospective cohort study using cystatin C- and creatinine-based eGFR
Source: PLoS One. 2026 Jul 27;21(7):e0329042. doi: 10.1371/journal.pone.0329042 (PMC13405090; doi:10.1371/journal.pone.0329042)
Supplement: S1 File — (PDF) [file pone.0329042.s001.pdf]

Malawi Epidemiology and intervention Research Unit (MEIRU)

KIDNEY HEALTH IN RURAL AND URBAN MALAWI (Impso Study)

MID-STREAM URINE SAMPLE PARTICIPANT INSTRUCTIONS

(ENGLISH)

You are invited to provide an early morning mid-stream urine sample as part of your participation in the Impso (Kidney Disease Progression) study.

It is crucial that the urine sample is collected aseptically according to the study instructions below. The study staff will go through these instructions with you now. If there you have any questions or there are any instructions you are unclear about, please ensure you clarify these with the study staff before providing the urine sample.

Please avoid sexual relations the night before the early morning urine sample collection.

Fieldworker to now read through mid-stream urine collection instructions with participant.

1. First wash your hands thoroughly with soap and water, then dry your hands.

2. When you are ready to provide the urine sample, carefully remove the lid of the urine collection pot and put the pot where you can easily reach it. IT IS VERY IMPORTANT that you do not touch anywhere on the inside of the pot with your hands or genitals

3. Cleanse your genital area with the cleaning wipe provided. Women should hold their folds of skin apart and clean between, from front to back. Men should pull back their foreskin, if applicable, and clean the head of their penis

4. Still holding the skin folds apart (women) or holding the foreskin back (men), start to pass urine into the toilet.

5. Pass a small amount of urine into the toilet

6. Then, mid-way through urination, move the urine collection pot under the path of your urine stream, and continue pass urine into the pot, filling the pot with urine until it is ½ (half) to ¾ (three quarters) full

7. Finish off by passing the remainder / final part of your urine into the toilet

8. Wash your hands again with soap and water

9. Carefully screw the lid tightly back on the urine collection pot and wrap/cover with the paper towels provided.

10. Carefully hand the urine pot back to the study staff, taking care not to touch or contaminate the inside, and taking care not to spill any

S1 File. Participant mid-stream urine sample collection instructions (English version)
