# Supplementary material for: Risk factors for decline in estimated glomerular filtration rate amongst Malawian adults living in rural Karonga: Protocol for a prospective cohort study using cystatin C- and creatinine-based eGFR
Source: PLoS One. 2026 Jul 27;21(7):e0329042. doi: 10.1371/journal.pone.0329042 (PMC13405090; doi:10.1371/journal.pone.0329042)
Supplement: S2 File — (.PDF) [file pone.0329042.s002.pdf]

S2 File. eGFR equations for use in planned analyses

Primary and secondary analyses

CKD-EPI 2012 equation for $eGFR_{cysC}$(1)

GFR = 133 x min(scysC/0·8, 1)$^{-0·499}$ x max(scysC/0·8, 1)$^{-1·328}$ x 0·996^{age} (x 0·932 if female)

min indicates the minimum of scysC/0·8 or 1 max indicates the maximum of scysC/0·8 or 1

CKD-EPI 2009 equation for $eGFR_{creat}$(2)

(without adjustment for African American ethnicity)

GFR = 141 × min(sCr/κ, 1)$^{\alpha}$ × max(sCr/κ, 1)$^{-1·209}$ × 0·993^{age} (× 1·018 if female) (× 1·159 if African American)

κ is 0·7mg/dl (62 µmol/L) for females and 0·9mg/dl (80 µmol/L) for males α is -0·329 for females and -0·411 for males
min indicates the minimum of sCr/κ or 1
max indicates the maximum of sCr/κ or 1

Sensitivity analyses

CKD-EPI 2021 equation for $eGFR_{creat}$(3)

GFR = 142 × min(sCr/κ, 1)$^{\alpha}$ × max(sCr/κ, 1)$^{-1·200}$ × 0·994^{age} (× 1·012 if female)

κ is 0·7mg/dl (62 µmol/L) for females and 0·9mg/dl (80 µmol/L) for males α is -0·241 for females and -0·302 for males
min indicates the minimum of sCr/κ or 1
max indicates the maximum of sCr/κ or 1

CKD-EPI 2021 combined equation for $eGFR_{cysC\text{-}creat}$(3)

GFR = 135 x min(sCr/κ, 1)$^{\alpha}$ x max(SCr/κ, 1)$^{-0·544}$ x min(scysC/0·8, 1)$^{-0·323}$ x max(scysC/0·8, 1) $^{-0·778}$ x 0·996^{age} (x 0·963 if female) (x 1·08 if African American)

κ is 0·7mg/dl (62 µmol/L) for females and 0·9mg/dl (80 µmol/L) for males α is -0·219 for females and -0·144 for males
min indicates the minimum of sCr/κ or 1
max indicates the maximum of sCr/κ or 1
min(scysC/0·8, 1) indicates the minimum of scysC/0·8 or 1 max(scysC/0·8,1) indicates the maximum of scysC/0·8 or 1

European Kidney Function Consortium (EFKC) equation for $eGFR_{cysC}$ (4,5)

Age	SCr/Q	Equation
18 to 40 years	< 1	$107{\cdot}3 \times (scysC/Q)^{-0{\cdot}322}$
	≥ 1	$107{\cdot}3 \times (scysC/Q)^{-1{\cdot}132}$
> 40 years	< 1	$107{\cdot}3 \times (scysC/Q)^{-0.322} \times 0{\cdot}990^{(Age - 40)}$
	≥ 1	$107{\cdot}3 \times (scysC/Q)^{-1{\cdot}132} \times 0{\cdot}990^{(Age - 40)}$

Q Values for cystatin C (in mg/L)
For ages 18 to 50 years:
0.83

For ages > 50 years:
0.83 + 0.005x(Age – 50)

European Kidney Function Consortium (EFKC) equation for $eGFR_{creat}$(5-7)

Age	SCr/Q	Equation
18 to 40 years	< 1	$107{\cdot}3 \times (SCr/Q)^{-0{\cdot}322}$
	≥ 1	$107{\cdot}3 \times (SCr/Q)^{-1{\cdot}132}$
> 40 years	< 1	$107{\cdot}3 \times (SCr/Q)^{-0.322} \times 0{\cdot}990^{(Age - 40)}$
	≥ 1	$107{\cdot}3 \times (SCr/Q)^{-1{\cdot}132} \times 0{\cdot}990^{(Age - 40)}$

Q Values
For ages 18 to 25 years:
Males:
$\ln(Q) = 3{\cdot}200 + 0{\cdot}259 \times Age - 0{\cdot}543 \times \ln(Age) - 0{\cdot}00763 \times Age^2 + 0{\cdot}0000790 \times Age^3$
Females:
$\ln(Q) = 3{\cdot}080 + 0{\cdot}177 \times Age - 0{\cdot}223 \times \ln(Age) - 0{\cdot}00596 \times Age^2 + 0{\cdot}0000686 \times Age^3$

For ages >25 years:
Males:
Q = 80 µmol/L (0·90 mg/dL) in White European populations(5); 85 µmol/L 0.96mg/dL in Black African populations(6,7)
Females:
Q = 62 µmol/L (0·70 mg/dL)) in White European populations(5);); 64 µmol/L 0.72mg/dL in Black African populations(6,7)
SCr and Q in µmol/L (to convert to mg/dL, divide by 88·4)
Q values (in µmol/L or mg/dL) correspond to the median SCr values for the age- and sex-specific populations.

Key

CKD-EPI = Chronic Kidney Disease Epidemiology CollaborationeGFR = estimated glomerular filtration rate; EKFC = European Kidney Function Consortium; SCr = serum creatinine; ; scysC = serum cystatin C;

The formula to convert serum creatinine form SI to conventional units (mg/L) is [serum creatinine/ x 88·4]. All equations are adjusted for body surface area (BSA) with units for GFR as $mL/min/1{\cdot}73m^2$.

References

1. Inker LA, Schmid CH, Tighiouart H, Eckfeldt JH, Feldman HI, Greene T, et al. Estimating glomerular filtration rate from serum creatinine and cystatin C. N Engl J Med. 2012;367(1):20-9.
2. Levey AS, Stevens LA, Schmid CH, Zhang YL, Castro AF, 3rd, Feldman HI, et al. A new equation to estimate glomerular filtration rate. Ann Intern Med. 2009;150(9):604-12.
3. Inker LA, Eneanya ND, Coresh J, Tighiouart H, Wang D, Sang Y, et al. New Creatinine- and Cystatin C-Based Equations to Estimate GFR without Race. N Engl J Med. 2021;385(19):1737-49.
4. Pottel H, Bjork J, Rule AD, Ebert N, Eriksen BO, Dubourg L, et al. Cystatin C-Based Equation to Estimate GFR without the Inclusion of Race and Sex. N Engl J Med. 2023;388(4):333-43.
5. Delanaye P, Cavalier E, Pottel H, Stehlé T. New and old GFR equations: a European perspective. Clin Kidney J. 2023 Mar 15;16(9):1375-1383. doi: 10.1093/ckj/sfad039. PMID: 37664574; PMCID: PMC10469124.
6. Pottel H, Bjork J, Courbebaisse M, Couzi L, Ebert N, Eriksen BO, et al. Development and Validation of a Modified Full Age Spectrum Creatinine-Based Equation to Estimate Glomerular Filtration Rate : A Cross-sectional Analysis of Pooled Data. Ann Intern Med. 2021;174(2):183-91.
7. Bukabau JB, Yayo E, Gnionsahé A et al. Performance of creatinine- or cystatin C–based equations to estimate glomerular filtration rate in sub-Saharan African popu- lations. Kidney Int 2019;95:1181–9.
