# Supplementary material for: Risk factors for decline in estimated glomerular filtration rate amongst Malawian adults living in rural Karonga: Protocol for a prospective cohort study using cystatin C- and creatinine-based eGFR
Source: PLoS One. 2026 Jul 27;21(7):e0329042. doi: 10.1371/journal.pone.0329042 (PMC13405090; doi:10.1371/journal.pone.0329042)
Supplement: S3 File — (PDF) [file pone.0329042.s003.pdf]

S3 File. Indications for referral for clinical assessment

Information source	Referral criteria
General clinical presentation	Presentation with acute physical or mental illness (e.g. fever, acute confusion)
Interviewer-led survey questions	Reported history of visible haematuria, not previously investigated and/or treated
	Reported history of significant medical condition (e.g. HIV, TB, Hepatitis B), not previously treated
	Reported urinary tract infection (UTI) symptoms, not previously reviewed by a clinician, or previously reviewed/treated but not improving
Household level HIV testing and counselling	Positive result
	Invalid or indeterminate result
Household level dipstick urinalysis	Visible haematuria
	Persistent (trace or above on two urine samples) non-visible haematuria
	Persistent (1+ or above on two urine samples) proteinuria
	Glucose ≥250mg/dL and no known diagnosis of diabetes
	Both glucose and ketones positive (trace or above)
	High ketones (≥40mg/dL) irrespective of dipstick glucose
	Bilirubin 1+ or above
	Urobilinogen ≥2mg/dL
Urine microscopy and gram stain	Schistosoma ova visualised
	Red cell casts visualised
	Positive gram stain if clinical symptoms/signs of urine infections also present
Serum sample biochemistry	$eGFR_{cysC} < 60$ ml/min/1.73m^2
