# Supplementary material for: Risk factors for decline in estimated glomerular filtration rate amongst Malawian adults living in rural Karonga: Protocol for a prospective cohort study using cystatin C- and creatinine-based eGFR
Source: PLoS One. 2026 Jul 27;21(7):e0329042. doi: 10.1371/journal.pone.0329042 (PMC13405090; doi:10.1371/journal.pone.0329042)
Supplement: S4 Table — (PDF) [file pone.0329042.s004.pdf]

S4 Table. Comparison of sociodemographic and clinical characteristics of adults aged ≥ 18 years living within the Karonga HDSS, with and without availability of cystatin C tested on stored serum samples

(n = 13,821).

**Characteristic,** at time of NCD survey (2013-16)	**Individuals with cystatin C tested** (n = 2792)	**Individuals without cystatin C tested** (n = 11,029)	**p value**
Sex (n, %)			
• Male	1230 (44.1)	4597 (41.7)	0.023
• Female	1562 (56.0)	6432 (58.3)	
Age (median, IQR)	35 (25 – 49)	34 (25 – 47)	0.003
Marital status (n, %)			
• Never married	306 (11.0)	1589 (14.4)	
• Married	2125 (76.1)	7580 (68.7)	0.000
• Divorced or separated	160 (5.7)	900 (8.2)	
• Widowed	192 (6.9)	948 (8.6)	
• Unknown	9 (0.3)	12 (0.1)	
Highest level of education (n, %)			
• No formal	151 (5.4)	444 (4.0)	
• Standard 1 – 5	404 (14.5)	1596 (14.5)	
• Standard 6 – 8	1487 (53.3)	4985 (45.2)	0.000
• Secondary	714 (25.6)	3789 (34.4)	
• Tertiary	36 (1.3)	215 (2.0)	
Occupation status (n, %)			
• Unemployed	28 (1.0)	348 (3.2)	
• At home, housework	76 (2.7)	757 (6.9)	
• Subsistence farming/fishing	2247 (80.5)	6386 (57.9)	
• Employee	74 (2.7)	649 (5.9)	0.000
• Self-employed	161 (5.8)	1731 (15.7)	
• Student	182 (6.5)	832 (7.5)	
• Retired	14 (0.5)	111 (1.0)	
• Other	10 (0.4)	215 (2.0)	
Smoking status (n, %)*			
• Never smoked	2551 (91.4)	9972 (90.4)	0.123
• Former or current smoker	241 (8.6)	1057 (9.6)	
Height, cm (median, IQR)^	158.6 (153.4 – 164.2)	159.1 (153.7 – 164.9)	0.005
Weight, kg (median, IQR)#	55.7 (50.3 – 61.5)	56.3 (50.6 – 63.1)	0.000
Body mass index, kg/m² (n, %)†			
• < 18	146 (5.2)	553 (5.0)	
• 18 to < 25	2091 (74.9)	7928 (71.9)	0.007
• 25 to < 30	355 (12.7)	1573 (14.3)	
• ≥ 30	108 (3.9)	523 (4.7)	
• Missing	92 (3.3)	452 (4.1)	
Hypertension, mmHg (n, %)‡			
• No	2445 (87.6)	9670 (87.7)	0.409
• Yes	343 (12.3)	1352 (12.3)	
• Missing	4 (0.1)	7 (0.1)	
Diabetes status (n, %)§			
• No	2694 (96.5)	9177 (83.2)	0.000
• Yes	98 (3.5)	110 (1.0)	
• Missing	0 (0.0)	1742 (15.8)	
HIV status, (n, %)¥			
• Non-reactive	1578 (56.5)	8161 (74.0)	0.000
• Reactive	189 (6.8)	979 (8.9)	
• Unknown	1025 (36.7)	1889 (17.1)	

p values for between group differences were calculated in Stata MP 18.0 using Pearson's chi-squared test for categorical variables and the Wilcoxon rank-sum test for non-normally distributed continuous variables.

*Smoking defined as use of either cigarettes or other tobacco products.

^Data available for n = 13,776, missing for n=45; 44 from the group without cystatin C tested and one from the group with cystatin C tested.

#Data available for n = 13,793, missing for n=28; all from the group without cystatin C tested.

†Data missing for 544 overall, of which 498 were not measured due to current pregnancy, 27 were not pregnant but are missing height and weight, 18 were not pregnant but are missing height only, and 1 was not pregnant but is missing weight only.

‡ Hypertension was measured in the NCD survey and was defined as blood pressure greater than 140/90mmHg, after taking three readings in total, five minutes apart, and averaging the 2nd and 3rd reading.

§ Diabetes status was ascertained through a combination of self-report and testing (fasting and/or random blood glucose) tested during the NCD survey.

¥HIV status is based on self-report data collected in the NCD survey.
