# Supplementary material for: Risk factors for decline in estimated glomerular filtration rate amongst Malawian adults living in rural Karonga: Protocol for a prospective cohort study using cystatin C- and creatinine-based eGFR
Source: PLoS One. 2026 Jul 27;21(7):e0329042. doi: 10.1371/journal.pone.0329042 (PMC13405090; doi:10.1371/journal.pone.0329042)
Supplement: S5 Table — (PDF) [file pone.0329042.s005.pdf]

S5 Table. Baseline sociodemographic and clinical characteristics of adults with cystatin C tested on stored serum samples, according to baseline $eGFR_{cysC}$
(n = 2792).

Characteristic*	Baseline $eGFR_{cysC}$ ≥ 90 ml/min/1.73m² (n = 1285)		Baseline $eGFR_{cysC}$ < 90 ml/min/1.73m² (n = 1507)		p value
Sex (n, %)					
• Male	481	(37.4)	749	(49.7)	0.000
• Female	804	(62.6)	758	(50.3)	
Age (median, IQR)	31	(24 – 40)	43	(30 – 58)	0.000
Marital status (n, %)*					
• Never married	162	(12.6)	144	(9.6)	
• Married	1007	(78.4)	1118	(74.2)	0.000
• Divorced or separated	70	(5.5)	90	(6.0)	
• Widowed	40	(3.1)	152	(10.1)	
• Unknown	6	(0.5)	3	(0.2)	
Highest level of education (n, %)					
• No formal	30	(2.3)	116	(7.7)	
• Standard 1 – 5	141	(11.0)	281	(18.7)	
• Standard 6 – 8	697	(54.2)	777	(51.6)	0.000
• Secondary	390	(30.4)	321	(21.3)	
• Tertiary	27	(2.1)	12	(0.8)	
Occupation status (n, %)					
• Unemployed	7	(0.5)	21	(1.4)	
• At home, housework	28	(2.2)	48	(3.2)	
• Subsistence farming/fishing	1021	(79.5)	1226	(81.4)	
• Employee	47	(3.7)	27	(1.8)	0.001
• Self-employed	84	(6.5)	77	(5.1)	
• Student	91	(7.1)	91	(6.0)	
• Retired	4	(0.3)	10	(0.7)	
• Other	3	(0.2)	7	(0.5)	
Smoking status (n, %)*					
• Never smoked	1198	(93.2)	1330	(88.3)	0.000
• Former or current smoker	87	(6.8)	177	(11.8)	
Height, cm (median, IQR)†	158.0	(153.1 – 164.0)	159.0	(153.5 – 164.5)	0.033
Weight, kg (median, IQR)	55.2	(49.7 – 61.4)	56.3	(50.9 – 62.4)	0.001
Body mass index, kg/m² (n, %)‡					
• < 18	52	(4.1)	93	(6.2)	
• 18 to < 25	979	(76.2)	1082	(71.8)	0.000
• 25 to < 30	157	(12.2)	224	(14.9)	
• ≥ 30	37	(2.9)	78	(5.2)	
• Missing	60	(4.7)	30	(2.0)	
Hypertension, mmHg (n, %)					
• No	1189	(92.5)	1254	(83.2)	0.000
• Yes	96	(7.5)	250	(16.6)	
• Missing	0	(0.0)	3	(0.2)	
Diabetes status (n, %)					
• No	1236	(96.2)	1452	(96.4)	0.820
• Yes	49	(3.8)	55	(3.7)	
HIV status, (n, %)¥					
• Non-reactive	902	(70.2)	888	(58.9)	0.000
• Reactive	76	(5.9)	116	(7.7)	
• Unknown	307	(23.9)	503	(33.4)	

p values for between group differences were calculated in Stata MP 18.0 using Pearson's chi-squared test for categorical variables and the Wilcoxon rank-sum test for non-normally distributed continuous variables. eGFRcysc is calculated using the CKD-EPI (2012) cystatin C equation.(63)

*All n=2792 individuals with cystatin C tested took part in the 2013-16 NCD survey; but only 503 took part in the 2018-19 ARK study. Of these 503, 497 had cystatin C tested on an ARK study serum sample and 6 had cystatin C tested on an earlier stored sample from the NCD survey. Overall 2295 of the samples tested for cystatin C were NCD survey samples and 497 were ARK study samples. Baseline characteristics are reported using data collected contemporaneously to the time of serum sampling for cystatin C testing (i.e. if the sample tested was collected in NCD survey, baseline characteristics also come from the NCD survey; if the sample tested was collected in the ARK study, baseline characteristics also come from the ARK survey). The exception is for marital status and occupation category, for which all data comes from the NCD study. Therefore for the 497 individuals who had cystatin C tested on serum samples collected in

the ARK study, it is possible marital status and occupation may have changed in the interim period between the time of the NCD survey (reported here), and the time of serum sampling for cystatin C testing.

†Height data available for n = 2791, missing for 1 person in the eGFR < 90 ml/min/1.73m^2 group

‡BMI data missing for n=90 in total, 60 from the eGFR ≥ 90 ml/min/1.73m^2 group and the 30 from the eGFR < 90 ml/min/1.73m^2 group. 89 of the 90 individuals missing BMI data were missing BMI due to be currently pregnant at the time of data collection. The remaining 1 individual (from the eGFR < 90 ml/min/1.73m^2 group) was missing height data.

¥HIV status is based on contemporaneous self-report data for individuals with cystatin C tested on samples collected in the NCD survey. For individuals with cystatin C tested on samples collected in the later ARK study, HIV status is based on contemporaneous HIV test data. However individuals with 'unknown' status in the ARK study (not tested) have been classified as 'positive' if the individual previously self-reported as 'positive' in the earlier NCD survey. Individuals with 'unknown' status in the ARK study who previously self-reported as negative remain in the unknown status category.
