# Supplementary material for: Risk factors for decline in estimated glomerular filtration rate amongst Malawian adults living in rural Karonga: Protocol for a prospective cohort study using cystatin C- and creatinine-based eGFR
Source: PLoS One. 2026 Jul 27;21(7):e0329042. doi: 10.1371/journal.pone.0329042 (PMC13405090; doi:10.1371/journal.pone.0329042)
Supplement: S6 File — (PDF) [file pone.0329042.s006.pdf]

S6 file. Reflexivity statement

Domain	Question	Response
Study conceptualization	1. How does this study address local research and policy priorities?	This study builds upon two large population based surveys of long-term health conditions in Malawi, and on the ARK study. The latter demonstrated that the prevalence of chronic kidney disease in African populations, including in Malawi, has been significantly underestimated by use of creatinine-based eGFR measures. The Malawi government recognises the growing public health threat posed by long-term health conditions in the country and is operationalising measures to implement to strengthen integrated health services, through its the Health Sector Strategic Plan III (2023-30). However there remains limited data on the kidney disease in Malawi, in particular a lack of data on long-term health outcomes and on the patient groups at highest risk for development and progression of CKD. This study will contribute important data which will help guide policy decision-making.
	2. How were local researchers involved in study design?	HCM, FL and CP were integral to the conceptualisation and design of CMS's overall PhD study, of which this study is one component, through their roles as PhD supervisors. AD and FK contributed to design and implementation of community sensitisation and engagement aspects of the study and provided orientation and guidance to the study team to ensure that research procedures took account of local setting and culture. AD, FK, SMM, TNG provided input and guidance on field data collection activities. CM contributed to design of laboratory standard operating procedures and CP to the design of clinical referral and assessment standard operating procedures. PK, BBM and DN contributed to design of data collection tools and management systems. DB and LK contributed to revision of Chitumbuka translations of data collection tools during the pilot phase to improve understandability (for example, or medical terminology) and cultural acceptability.
Research management	3. How has funding been used to support the local research team(s)?	Funds from the grant for this study have supported the local research team through salaries, training, equipment provision and support to laboratory and data office running costs.
Data acquisition and analysis	4. How are research staff who conducted data collection acknowledged?	Research staff who conducted data collection for the early pilot work described in this manuscript are listed as authors.
	5. How have members of the research partnership been provided with access to study data?	As this is a protocol paper the data collection is not yet complete, however updates on recruitment progress are shared with members of the research partnership on a regular basis. On completion of the study, data will be retained on secure MEIRU servers in Malawi, accessible to immediate team members in Malawi or via secure transfer to team members in the UK. Access to preliminary or final analytical datasets for people outside of the core study team will be granted on request by the MEIRU data access committee, conditional on meeting standardised procedures.
	6. How were data used to develop analytical skills within the partnership?	As this is a study protocol, analysis has not yet taken place for current study. However several team members have been supported by the wider partnership in development of analytical skills through their involvement in the linked studies (NCD survey, HLM LTC survey and ARK study) that precede this work.
Data interpretation	7. How have research partners collaborated in interpreting study data?	As this is a study protocol, interpretation of study data has not yet taken place.

Drafting and revising for intellectual content	8. How were research partners supported to develop writing skills?	The initial draft of the manuscript was written by CMS but all available co-authors except LK were involved in revisions and approved the final version of the manuscript.
	9. How will research products be shared to address local needs?	The results of this research will be shared with the communities involved through well-established community engagement mechanisms, including engagement with a community advisory committee, and with local researchers in Malawi, through forums such as the Kamuzu University of Health Sciences Research Dissemination Conference. Importantly the study findings will also be shared with Ministry of Health Policy makers: an initial overview of the study aims and methods has already been presented at a Non-Communicable Diseases Technical Working Group meeting held in March 2025.
Authorship	10.How is the leadership, contribution and ownership of this work by LMIC researchers recognised within the authorship?	All LMIC researchers who played a leading role in this work are listed as co-authors, and others who supported the work are acknowledged. We recognise the important contribution of all MEIRU staff to the day to day operations of the Health and Demographic Surveillance Site and larger studies linked to this work, without which this study would not be possible.
	11. How have early career researchers across the partnership been included within the authorship team?	Several early career researchers based in Malawi are included within the authorship team, having been involved in both research and writing stages of this manuscript.
	12. How has gender balance been addressed within the authorship?	Six authors are female and 14 authors are male, reflecting the contributions of individuals who have been actively involved in this research.
Training	13. How has the project contributed to training of LMIC researchers?	This study forms part of a wider program of work at MEIRU where researchers working across linked studies meet on a fortnightly basis to share updates and learning. Direct training through this study has been provided to research nurses, medical and clinical officers covering Good Clinical Practice, study procedures, assessment and management of kidney disease. Through the PhD which this study forms a part of, CMS also participates in regular peer-led PhD student meetings at the Malawi-Liverpool Wellcome Research Programme, a forum which provides an opportunity for both Malawian and international PhD students to develop presentation skills and exchange peer-to-peer feedback.
Infrastructure	14. How has the project contributed to improvements in local infrastructure	This project has not directly contributed to improvements in local infrastructure. However, through the linked studies that preceded this work, a non-communicable diseases and mental health clinic have been established at Chilumba Rural Hospital, at which study participants requiring medical assessment are reviewed.
	15. What safeguarding procedures were used to protect local study participants and researchers?	Full research ethics approval was obtained for this study from both the National Health Sciences Research Committee in Malawi, and the Liverpool School of Tropical Medicine. Safeguarding procedures follow those outlined in the MEIRU Safeguarding Policy which was already established and operational prior to initiation of this study. Information on safeguarding procedures is included in the study participant information sheet, in language formats (Chitumbuka, Chichewa and English), including local contact details for participants to report and safeguarding concerns.
