# Supplementary material for: Risk factors for decline in estimated glomerular filtration rate amongst Malawian adults living in rural Karonga: Protocol for a prospective cohort study using cystatin C- and creatinine-based eGFR
Source: PLoS One. 2026 Jul 27;21(7):e0329042. doi: 10.1371/journal.pone.0329042 (PMC13405090; doi:10.1371/journal.pone.0329042)
Supplement: S7 File — (PDF) [file pone.0329042.s007.pdf]

S7 File. STROBE Statement—checklist of items that should be included in reports of observational studies

	Item No.	Recommendation	Page No.	Relevant text from manuscript
**Title and abstract**	1	(*a*) Indicate the study's design with a commonly used term in the title or the abstract	1 – 4	**Sections:** Title, Abstract
		(*b*) Provide in the abstract an informative and balanced summary of what was done and what was found	3	**Section:** Abstract (Methods)
**Introduction**				
Background/rationale	2	Explain the scientific background and rationale for the investigation being reported	5 – 7	**Section:** Introduction
Objectives	3	State specific objectives, including any prespecified hypotheses	7	**Section:** Introduction (Aims and Objectives)
**Methods**				
Study design	4	Present key elements of study design early in the paper	8 – 9	**Section:** Methods (Study Design and Setting)
Setting	5	Describe the setting, locations, and relevant dates, including periods of recruitment, exposure, follow-up, and data collection	8 – 17	**Section:** Methods (Study Design and Setting, Recruitment and Consent, Household-level Data Collection, Pilot Phase) and Fig 1
Participants	6	(*a*) *Cohort study*—Give the eligibility criteria, and the sources and methods of selection of participants. Describe methods of follow-up<br>*Case-control study*—Give the eligibility criteria, and the sources and methods of case ascertainment and control selection. Give the rationale for the choice of cases and controls<br>*Cross-sectional study*—Give the eligibility criteria, and the sources and methods of selection of participants	9 – 14	**Sections:** Methods (Participants, Recruitment and Consent, Household-level Data Collection), Fig 2 and Fig 3.
		(*b*) *Cohort study*—For matched studies, give matching criteria and number of exposed and unexposed<br>*Case-control study*—For matched studies, give matching criteria and the number of controls per case	n/a	Not applicable

Variables	7	Clearly define all outcomes, exposures, predictors, potential confounders, and effect modifiers. Give diagnostic criteria, if applicable	18 – 21	**Section:** Analysis (eGFR equations, Outcome Measures, Statistical Analysis,)
Data sources/ measurement	8*	For each variable of interest, give sources of data and details of methods of assessment (measurement). Describe comparability of assessment methods if there is more than one group	11 – 14<br>19 – 20	**Sections:** Methods (Household-level Data Collection, Laboratory Methods); Analysis (Outcome Measures)
Bias	9	Describe any efforts to address potential sources of bias	10 – 11<br>23 – 28	**Sections:** Methods (Recruitment and Consent); Discussion (including strengths and limitations)
Study size	10	Explain how the study size was arrived at	18	**Section:** Analysis (Sample Size)

Continued on next page

Quantitative variables	11	Explain how quantitative variables were handled in the analyses. If applicable, describe which groupings were chosen and why	20 – 21	**Section:** Analysis (Statistical Analysis)
Statistical methods	12	(*a*) Describe all statistical methods, including those used to control for confounding	20 – 21	**Section:** Analysis (Statistical Analysis)
		(*b*) Describe any methods used to examine subgroups and interactions	20 – 21	**Section:** Analysis (Statistical Analysis)
		(*c*) Explain how missing data were addressed	n/a	Not applicable; study ongoing and not anticipating missing data.
		(*d*) *Cohort study*—If applicable, explain how loss to follow-up was addressed<br>*Case-control study*—If applicable, explain how matching of cases and controls was addressed<br>*Cross-sectional study*—If applicable, describe analytical methods taking account of sampling strategy	n/a	Not applicable; study ongoing and not anticipating loss to follow-up.
		(*e*) Describe any sensitivity analyses	18 - 19	**Section:** Analysis (eGFR equations)
**Results**				
Participants	13*	(a) Report numbers of individuals at each stage of study—e.g. numbers potentially eligible, examined for eligibility, confirmed eligible, included in the study, completing follow-up, and analysed	n/a	Not applicable; as this is a study protocol, and recruitment is not yet complete. To be reported in final study report.
		(b) Give reasons for non-participation at each stage	n/a	Not applicable; as this is a study protocol, and recruitment is not yet complete. To be reported in final study report.
		(c) Consider use of a flow diagram	n/a	Not applicable; as this is a study protocol, and recruitment is not yet complete. To be reported in final study report.
Descriptive data	14*	(a) Give characteristics of study participants (e.g. demographic, clinical, social) and information on exposures and potential confounders	S4, S5	Not applicable; as this is a study protocol, and recruitment is not yet complete. However characteristics of individuals with baseline cystatin

				C available have been reported in supplementary file S3, and a comparison of individuals with baseline eGFRcysc <90 and ≥90 is shown in supplementary file S4
		(b) Indicate number of participants with missing data for each variable of interest	n/a	Not applicable; as this is a study protocol, and recruitment is not yet complete. To be reported in final study report.
		(c) *Cohort study*—Summarise follow-up time (e.g., average and total amount)	n/a	Not applicable; as this is a study protocol, and recruitment is not yet complete. To be reported in final study report.
Outcome data	15*	*Cohort study*—Report numbers of outcome events or summary measures over time	n/a	Not applicable; as this is a study protocol, and recruitment is not yet complete. To be reported in final study report.
		Case-control study—Report numbers in each exposure category, or summary measures of exposure	n/a	This is a cohort study protocol.
		Cross-sectional study—Report numbers of outcome events or summary measures	n/a	This is a cohort study protocol.
Main results	16	(*a*) Give unadjusted estimates and, if applicable, confounder-adjusted estimates and their precision (eg, 95% confidence interval). Make clear which confounders were adjusted for and why they were included	n/a	Not applicable; as this is a study protocol, and recruitment is not yet complete. Results to be reported in final study report.
		(*b*) Report category boundaries when continuous variables were categorized	n/a	Not applicable; as this is a study protocol, and recruitment is not yet complete. Results to be reported in final study report.
		(*c*) If relevant, consider translating estimates of relative risk into absolute risk for a meaningful time period	n/a	Not applicable; as this is a study protocol, and recruitment is not yet complete. Results to be reported in final study report.

Continued on next page

Other analyses	17	Report other analyses done—eg analyses of subgroups and interactions, and sensitivity analyses	n/a	Not applicable; as this is a study protocol, and recruitment is not yet complete. Results to be reported in final study report.
**Discussion**				
Key results	18	Summarise key results with reference to study objectives	n/a	Not applicable; as this is a study protocol, and recruitment is not yet complete. Results to be reported in final study report.
Limitations	19	Discuss limitations of the study, taking into account sources of potential bias or imprecision. Discuss both direction and magnitude of any potential bias	26 – 28	**Section:** Discussion (section on limitations)
Interpretation	20	Give a cautious overall interpretation of results considering objectives, limitations, multiplicity of analyses, results from similar studies, and other relevant evidence	n/a	Not applicable; as this is a study protocol, and recruitment is not yet complete. Results and their interpretation to be reported in final study report.
Generalisability	21	Discuss the generalisability (external validity) of the study results	n/a	Not applicable; as this is a study protocol, and recruitment is not yet complete. Results and their interpretation to be reported in final study report.
**Other information**				
Funding	22	Give the source of funding and the role of the funders for the present study and, if applicable, for the original study on which the present article is based	29	**Section:** Funding

*Give information separately for cases and controls in case-control studies and, if applicable, for exposed and unexposed groups in cohort and cross-sectional studies.

Note: An Explanation and Elaboration article discusses each checklist item and gives methodological background and published examples of transparent reporting. The STROBE checklist is best used in conjunction with this article (freely available on the Web sites of PLoS Medicine at http://www.plosmedicine.org/, Annals of Internal Medicine at http://www.annals.org/, and Epidemiology at http://www.epidem.com/). Information on the STROBE Initiative is available at www.strobe-statement.org.
