# Supplementary material for: Risk factors for decline in estimated glomerular filtration rate amongst Malawian adults living in rural Karonga: Protocol for a prospective cohort study using cystatin C- and creatinine-based eGFR
Source: PLoS One. 2026 Jul 27;21(7):e0329042. doi: 10.1371/journal.pone.0329042 (PMC13405090; doi:10.1371/journal.pone.0329042)
Supplement: S8 File — (PDF) [file pone.0329042.s008.pdf]

Inclusivity in global research

PLOS' policy on inclusivity in global research aims to improve transparency in the reporting of research performed outside of researchers' own country or community and ensures that PLOS publications reporting global research adhere to high standards for research ethics and authorship. Authors of relevant research articles may be asked to complete the questionnaire below, which outlines ethical, cultural, and scientific considerations specific to inclusivity in global research. This questionnaire may be requested when researchers have travelled to a different country to conduct research, if research uses samples collected in another country, research with Indigenous populations or their lands, or if research is on cultural artefacts. Researchers travelling to another country solely to use laboratory equipment will not normally be required to complete the questionnaire. However, the questionnaire can be requested at the journal's discretion for any submission – if you have been requested to complete this questionnaire by the PLOS journal you submitted to, please do so.

Please complete the questionnaire below and include this as a Supporting Information file with your manuscript. Note that if your paper is accepted for publication, this checklist will be published with your article in the supporting information files. Please ensure that you reference the checklist in the main body of your manuscript. We suggest adding a subsection 'Inclusivity in global research' to your Methods section and adding the following sentence: "Additional information regarding the ethical, cultural, and scientific considerations specific to inclusivity in global research is included in the Supporting Information (SX Checklist)"

The questions have been designed to be applicable to a wide range of study types, and there are subsections for both human subjects research and non-human subjects research. If any of the questions are not relevant to your research please mark them as "N/A" as appropriate.

Ethical considerations, permits and authorship

This section is applicable to all research types.

Provide details as to who granted permissions and/or consent for the study to take place in the Methods section of your manuscript. This should include the names of **all** ethics boards, governmental organizations, community leaders or other bodies that provided approval for the study. If individuals provided approval refer to these people by their role or title but do not list their name(s).

Reported on page number: 22

If there were any deviations from the study protocol after approval was obtained please provide details of these changes in the Methods section of your manuscript.

Reported on page number: Not applicable; there were no deviations from the study protocol afeter approval was obtained.

Did this study involve local collaborators that are residents of the country where the research was conducted or members of the community studied? If you do not have any authors from said communities, please provide an explanation for this below.

Yes, local collaborators were involved in all steps of the study from conceptualization through to writing. Full details of how local researchers were involved are outlines **in S6 File, Reflexivity Statement**, and all author contributions are detailed in the Author Contribution Statement.

Everyone listed as an author should meet PLOS' criteria for authorship and all individuals who meet these criteria should be included in the author byline, rather than the acknowledgements. For further information please see the journal's Authorship Policy.

Human subjects research (e.g. health research, medical research, cross-cultural psychology)

Did you obtain written informed consent from a representative of the local community or region before the research took place? How did you establish who speaks for the community? Details of written informed consent obtained from study participants should be reported separately in the Methods section of your manuscript.

Heads of all households within the Karonga Health and Demographic Surveillance Site (HDSS) provide written informed consent for participation in HDSS activities, which also includes providing consent to be approached about participation in health-related research studies. Written consent for participation in the HDSS is actively renewed on an annual basis but households can withdraw at any point they choose.

This study is linked to the overarching Healthy Lives Malawi (HLM) long-term conditions survey, for which a large community engagement program was aleady in place. All participants of the current study had provided written, informed consent to take part in the HLM survey. Prior to that, after first engaging with village heads, a series of open community meetings had taken place in all areas of the HDSS to provide information and a forum for discussion about the overall HLM survey as well as its linked studies.

Prior to initiation of this specific study, and prior to seeking National level Research Ethics Committee approval in Malawi, written local approval was obtained from the Karonga District Health Office. Following that, a meeting was held between the study team and the Karonga Area Development Committee (ADC). This committee consisted of of high level leaders with roles in the Chilumba area of jurisdiction where the study was due to take place, including senior group village headmen from across the area, government extension workers and line ministry representatives (e.g. from education, farming, forestry), faith leaders, as well as staff from non-government organisations (NGOs) and other community organisations. During the meeting, information was provided about the study aims and activities, including distribution of the study information sheets and informed consent forms for committee members to read. This was followed by a discussion session to answer questions and seek guidance and feedback on the planned study delivery. An attendance list was documented, and verbal consent and support for the study from the ADC was obtained prior to initiation of any study activities.

After commencement of the study, prior to initiation in each geographical area of the HDSS, meetings were held with village heads and community repesentatives (volunteers) from that area, to again provide information about the study and its activities, read through the information sheets in detail, and provide a forum for questions and discussion. Verbal consent and support for the study from the village head and community representatives was obtained prior to initiation of any study activities in each area.

How did members of the local community provide input on the aims of the research investigation, its methodology, and its anticipated outcome(s)?

The study investigator and authorship team includes fourteen Malawian researchers of whom five are specifically members of communities from the Karonga or Chilumba area where this study is based. These study team members contributed to the design and implementation of the study and the writing of this manuscript, as outlined in full in S6 File, Reflexivity Statement, and in the Author Contribution Statement. Local co-authors will also contribute to analysis of outcomes once the study recruitment is complete.

Wider members of the local community provided input on the aims, methodology and data collection methods through the established HLM community engagement meeting programme, and through the meetings with the Area Development Committee (ADC), village heads and community representatives (as described on the previous page). During the ADC meeting, the study was welcomed positively, with feedback including the need for future studies to include even larger numbers of participants including expansion beyond the current HDSS area. Feedback from the ADC highlighted the need for ongoing community sensitization and guided format and implementation of the community engagement meetigns with village heads and volunteers which have continued throughout the study prior to its initiation in each geographical area (see previous page). Other important considerations raised through meetings these community groups concerned the need to ensure communication of study results to research participants, and provision of clinical care to inidivuals identified to have kidney disease or other clinical problems. These considerations were aigned with the study implementation, including design of the standard operating procedures for return of study results to participants and clinical review of participants identified to have possible kidney disease. The latter is provided by MEIRU clinicians through a non-communicable disease (NCD) clinic set up at Chilumba Rural Hospital by the wider HLM study to support clinical care for the local population.

When engaging with the local community, how did you ensure that the informed consent documents and other materials could be understood by local stakeholders?

All participant-facing study documents including the participant information sheets, informed consent forms and all data collection tools were provided in Chitumbuka and Chichewa.

The original versions of these documents and data collection tools, which had been translated from English into Chitumbuka and Chichewa, underwent a thorough internal pilot and back-translation exercise by two of the local, medically-trained study team members (DB and LK). During this process, translations and questionnaire questions were revised to improve understandability and cultural acceptability, including the understandability of terminlology related to medical conditions. Following the external (community-based) pilot phase, further revisions were made based on feedback and observations in the field (see Methods; Pilot Phase).

In all engagement activities and meetings, copies of the study documents were provided in these local languages but the study team also read through all the study information aloud. Question and answer sessions enabled clarifications to be sought and addressed.

Will the findings of the research be made available in an understandable format to stakeholders in the community where the study was conducted (e.g. via a presentation, summary report, copies of publications, etc.)? Please provide details of how this will be achieved.

Once the study analysis is complete, meetings will be held to share the study findings with all relevant stakeholders including the Karonga District Health Office, the Karonga Area Development Committee, the recently formed Community Advisory group, and with community groups in the areas where the study took place.

Written summary reports will be also provided, and copies of full publications and/or abstracts as appropriate, with abstracts and summary reports translated in Chitumbuka and Chichewa.

Local media will also be engaged to assist with wider dissemination of study findings including via the radio.

Non-human subjects research using specimens/ animals collected as part of the study, or those housed in archival collections. Examples include archaeology, paleontology, botany and zoology.

Did the permission you obtained from a local authority to perform the study include an agreement on access to outputs and benefit sharing? This may include procedures to enable fair distribution of the benefits and resources arising from the research performed. Please include any details of Prior Informed Consent and Benefit Sharing Agreements obtained. These may be required by field-specific regulations, for example the Convention on Biological Diversity (CBD) and the associated Nagoya Protocol.

Not applicable.

If the material used in your study was imported, please A) provide the year it was imported and B) indicate whether permits were obtained to import/export the materials used, C) provide details of any permits obtained. If this information is not available, please indicate this.

Not applicable.

If you used archival specimens, please state how the material used in your study was acquired by the institute it is held in and provide details of any permits obtained for the original excavations/ sample collection. If this information is not available, please indicate this.

Not applicable.

How was the potential cultural significance of the materials collected in your study to local communities considered in your research design? Were Indigenous peoples and/or local researchers and institutions involved with archaeological excavations / collection of specimens? If so, please provide a description of their involvement.

Not applicable.

If your manuscript includes photographs of human remains please indicate whether authors obtained permission from descendants or affiliated cultural communities to do so.

Not applicable.
