# Supplementary material for: Risk factors for decline in estimated glomerular filtration rate amongst Malawian adults living in rural Karonga: Protocol for a prospective cohort study using cystatin C- and creatinine-based eGFR
Source: PLoS One. 2026 Jul 27;21(7):e0329042. doi: 10.1371/journal.pone.0329042 (PMC13405090; doi:10.1371/journal.pone.0329042)
Supplement: Attachment — Submitted filename: Response to reviewers.docx [file pone.0329042.s011.docx]

**PONE-D-25-34787: Response to reviewers**

**Editor comments and journal requirements**

1. **Please ensure that your manuscript meets PLOS ONE's style requirements, including those for file naming. The PLOS ONE style templates can be found at** https://journals.plos.org/plosone/s/file?id=wjVg/PLOSOne_formatting_sample_main_body.pdf **and** https://journals.plos.org/plosone/s/file?id=ba62/PLOSOne_formatting_sample_title_authors_affiliations.pdf

Response:

We have double checked all the submitted documents and can confirm the resubmitted documents meet the PLOS ONE style requirements including those for file naming and formatting.

2. **Please include a complete copy of PLOS’ questionnaire on inclusivity in global research in your revised manuscript. Our policy for research in this area aims to improve transparency in the reporting of research performed outside of researchers’ own country or community. The policy applies to researchers who have travelled to a different country to conduct research, research with Indigenous populations or their lands, and research on cultural artefacts. The questionnaire can also be requested at the journal’s discretion for any other submissions, even if these conditions are not met.  Please find more information on the policy and a link to download a blank copy of the questionnaire here: https://journals.plos.org/plosone/s/best-practices-in-research-reporting. Please upload a completed version of your questionnaire as Supporting Information when you resubmit your manuscript.**

Response:

Thank you very much, we have now completed this documented and uploaded it under Supporting Information.

3. **We note that your Data Availability Statement is currently as follows: [All relevant data are within the manuscript and its Supporting Information files.] Please confirm at this time whether or not your submission contains all raw data required to replicate the results of your study. Authors must share the “minimal data set” for their submission. PLOS defines the minimal data set to consist of the data required to replicate all study findings reported in the article, as well as related metadata and methods** (https://journals.plos.org/plosone/s/data-availability#loc-minimal-data-set-definition).

**For example, authors should submit the following data:**

**- The values behind the means, standard deviations and other measures reported;**

**- The values used to build graphs;**

**- The points extracted from images for analysis.**

**Authors do not need to submit their entire data set if only a portion of the data was used in the reported study.**

**If your submission does not contain these data, please either upload them as Supporting Information files or deposit them to a stable, public repository and provide us with the relevant URLs, DOIs, or accession numbers. For a list of recommended repositories, please see** https://journals.plos.org/plosone/s/recommended-repositories

**If there are ethical or legal restrictions on sharing a de-identified data set, please explain them in detail (e.g., data contain potentially sensitive information, data are owned by a third-party organization, etc.) and who has imposed them (e.g., an ethics committee). Please also provide contact information for a data access committee, ethics committee, or other institutional body to which data requests may be sent. If data are owned by a third party, please indicate how others may request data access.**

Response:

Only a portion of the data from the MEIRU NCD survey and the African Research on Kidney Disease (ARK) Consortium study was used to create the tables in Supplementary files S4 and S5 submitted with this protocol paper (these files were previously named S3 and S4 but have been updated to S4 and S5 due to addition of new supplementary file S2 in response to reviewer 1 comments). No pilot data or outcome data from the study is reported within the main article.

We have not submitted the full datasets but have revised our Data Availability Statement and provide clarity on how readers can access the raw data.

*Data from both these studies is publicly available. The full data from the MEIRU NCD survey can be accessed via the following public repository;* [*https://datacompass.lshtm.ac.uk/id/eprint/3834/*](https://url.uk.m.mimecastprotect.com/s/mp60CMJPASkVwj2CwfLs8aA3C?domain=datacompass.lshtm.ac.uk/)*; with some creatinine data available in an additional public repository;* [*https://datacompass.lshtm.ac.uk/id/eprint/1150/*](https://url.uk.m.mimecastprotect.com/s/3NxxCNYgBSP5OoZF4hGsyK51F?domain=datacompass.lshtm.ac.uk/)

*Data from the ARK study is available publicly at:* [*https://github.com/ARKconsortium/iohexol_mGFR_eGFR*](https://url.uk.m.mimecastprotect.com/s/TM2GCMJPASkWomLs1uLs8Vd1T?domain=github.com)*.*

*This is a standardised set of relevant data from all three study sites involved in that study: Malawi, South Africa and Uganda. The dataset includes deidentified individual participant data related to estimated and measured GFR, with accompanying analytic code used for development of a new creatinine-based GFR-estimating equation.*

*The specific merged dataset used to create supplementary tables S4 and S5 used anonymised participant-level data from both the MEIRU NCD survey and the Malawi arm of the ARK study (all individuals had taken part in the MEIRU NCD survey; a subset had additional taken part in the ARK study). This data will be publicly available by the time the currently described study is completed and published, but is not yet accessible in a public repository as work is ongoing to finalise open datasets. However while this work is ongoing the dataset can be accessed on request through application to the MEIRU data access committee (*[*mia.crampin@glasgow.ac.uk*](mailto:mia.crampin@glasgow.ac.uk)*;* [*paul.kambiya@meiru.mw*](mailto:paul.kambiya@meiru.mw)*). Deidentified datasets from the currently ongoing Impso study, described in this protocol paper, will also be made publicly available once analysis is complete.*

4. **Your ethics statement should only appear in the Methods section of your manuscript. If your ethics statement is written in any section besides the Methods, please move it to the Methods section and delete it from any other section. Please ensure that your ethics statement is included in your manuscript, as the ethics statement entered into the online submission form will not be published alongside your manuscript.**

Response:

We have doubled checked and confirm that the ethics statement only appeared in the Methods section of our Manuscript, under the Methods subsection, “Ethics, Governance and Regulations; Ethical approval” – please see **page 21-22, lines 468-486**. We have amended the subheading from “ethical approval” to “ethical approval and informed consent” and added an additional statement on informed consent. The specific details of the informed consent process are outlined in the earlier Methods subsection, “Recruitment and consent” (**pages 11-12, lines 211 - 226**).

5. **We note that Figure 1 in your submission contain [map/satellite] images which may be copyrighted. All PLOS content is published under the Creative Commons Attribution License (CC BY 4.0), which means that the manuscript, images, and Supporting Information files will be freely available online, and any third party is permitted to access, download, copy, distribute, and use these materials in any way, even commercially, with proper attribution. For these reasons, we cannot publish previously copyrighted maps or satellite images created using proprietary data, such as Google software (Google Maps, Street View, and Earth). For more information, see our copyright guidelines:** [http://journals.plos.org/plosone/s/licenses-and-copyright](https://url.uk.m.mimecastprotect.com/s/9ZOdC5Rk4CpOAmzIjSXskSEIl?domain=journals.plos.org).

**We require you to either (1) present written permission from the copyright holder to publish these figures specifically under the CC BY 4.0 license, or (2) remove the figures from your submission:**

**1. You may seek permission from the original copyright holder of Figure 1 to publish the content specifically under the CC BY 4.0 license.**

**We recommend that you contact the original copyright holder with the Content Permission Form** ([http://journals.plos.org/plosone/s/file?id=7c09/content-permission-form.pdf](https://url.uk.m.mimecastprotect.com/s/Ftx-C6BlNH0xX2gclTxs5pUOe?domain=journals.plos.org)) **and the following text:**

**“I request permission for the open-access journal PLOS ONE to publish XXX under the Creative Commons Attribution License (CCAL) CC BY 4.0** ([http://creativecommons.org/licenses/by/4.0/](https://url.uk.m.mimecastprotect.com/s/-EBnC7pm0iV9L50CEUGsohRWS?domain=creativecommons.org)). **Please be aware that this license allows unrestricted use and distribution, even commercially, by third parties. Please reply and provide explicit written permission to publish XXX under a CC BY license and complete the attached form.”**

**Please upload the completed Content Permission Form or other proof of granted permissions as an "Other" file with your submission.**

**In the figure caption of the copyrighted figure, please include the following text: “Reprinted from [ref] under a CC BY license, with permission from [name of publisher], original copyright [original copyright year].”**

**2. If you are unable to obtain permission from the original copyright holder to publish these figures under the CC BY 4.0 license or if the copyright holder’s requirements are incompatible with the CC BY 4.0 license, please either i) remove the figure or ii) supply a replacement figure that complies with the CC BY 4.0 license. Please check copyright information on all replacement figures and update the figure caption with source information. If applicable, please specify in the figure caption text when a figure is similar but not identical to the original image and is therefore for illustrative purposes only.**

**The following resources for replacing copyrighted map figures may be helpful**

**USGS National Map Viewer (public domain):** [http://viewer.nationalmap.gov/viewer/](https://url.uk.m.mimecastprotect.com/s/ZZtaC8MnNiPx9VAcgc7sy9EAr?domain=viewer.nationalmap.gov)

**The Gateway to Astronaut Photography of Earth (public domain):** [http://eol.jsc.nasa.gov/sseop/clickmap/](https://url.uk.m.mimecastprotect.com/s/3e84C9DoNfNP8y6cKfzsqzjog?domain=eol.jsc.nasa.gov)

**Maps at the CIA (public domain):** [https://www.cia.gov/library/publications/the-world-factbook/index.html](https://url.uk.m.mimecastprotect.com/s/d8W8C00N7SgljO9uRh1s9jGVT?domain=cia.gov) **and** [https://www.cia.gov/library/publications/cia-maps-publications/index.html](https://url.uk.m.mimecastprotect.com/s/5UT2CgnOESwn8XjuYixs45ifC?domain=cia.gov)

**NASA Earth Observatory (public domain):** [http://earthobservatory.nasa.gov/](https://url.uk.m.mimecastprotect.com/s/YaL3Cj0jKSGkgLws4s3smpXCN?domain=earthobservatory.nasa.gov)

**Landsat:** [http://landsat.visibleearth.nasa.gov/](https://url.uk.m.mimecastprotect.com/s/0kIECkrkLiXZgVRCxtEsG4kyy?domain=landsat.visibleearth.nasa.gov)

**USGS EROS (Earth Resources Observatory and Science (EROS) Center) (public domain):** [http://eros.usgs.gov/#](https://url.uk.m.mimecastprotect.com/s/No-bCl2lgUPDLJ3cmuvszUAJe?domain=eros.usgs.gov)

**Natural Earth (public domain):** [http://www.naturalearthdata.com/](https://url.uk.m.mimecastprotect.com/s/QcH_CmYmjSPGvw7cPC5sRw3TK?domain=naturalearthdata.com)

Response:

We have amended the image in Figure 1 to just include just the zoomed in map of the Karonga Health and Demographic Surveillance Site. One of the authors of this manuscript, AD, generated this map presented in Figure 1 using Quantum Geographic Information System (QGIS) a free and open-source software that allows users to create, edit, visualize, analyse, and publish geospatial information. From 2002 to 2005, GPS units were used by project officers and village motivators to track village and cluster boundaries.(1) Since 2005 to date more cluster boundary data has been collected and stored by the project. We loaded this project data into QGIS 3.32 Lima in order to generate the map. The QGIS user guidance states that:

*“There is no requirement to mention QGIS for maps produced with it. It is of course really welcome if you would like to add a note saying that the map was produced with QGIS. “Made with QGIS” or “Map created using the Free and Open Source QGIS” are good examples of such a note. Do not say ©QGIS as QGIS does not hold copyrights on your work”.*

This guidance can be found here:

<https://qgis.org/resources/support/faq/>

We have therefore amended the caption of Figure 1 to include the QGIS-endorsed statement:

*Map created using the Free and Open Source Quantum Geographic Information System (QGIS):* [*https://qgis.org/*](https://qgis.org/)

Since QGIS state they do not hold copyrights on images created using their software, we could not ask them to complete the Content Permission Form, however please do let us know if we need to provide any further information or clarification with regards to this image.

6. **If the reviewer comments include a recommendation to cite specific previously published works, please review and evaluate these publications to determine whether they are relevant and should be cited. There is no requirement to cite these works unless the editor has indicated otherwise.**

Response:

Not applicable; the reviewer comments did not include any recommendations to cite specific works.

**Reviewer comments**

**Reviewer 1**

**Authors teamed up with Malawian associates and planned to develop a study system to help clarify the CKD prevalence and risk factors of kidney function decline. This manuscript is descriptive work plan worth encouraging. I had experience to work in that country and understand how much efforts would be necessary to overcome the gap between contemporary medical system and that in Malawi. Comments are as the followings.**

Thank you very much for the positive and encouraging feedback. All co-authors, Malawian and international, are full team members of the study, which aims to investigate risk factors for kidney function decline (see Introduction; Aims and Objectives, **page 8 lines 127-131**).

1. **Readers would like to understand why authors suggested that CRTN-based eGFR is not so suitable in Africans.**

Response

We have outlined the background as to why creatinine-based eGFR performs poorly compared to cystatin C and gold standard measured GFR in the Please see Introduction (**page 5, lines 67 to 70**) which outlines the background regarding the difference in performance between creatinine-based eGFR and gold standard measured GFR in African populations:

*“Importantly, the most readily available creatinine-based equations for estimating glomerular filtration rate (eGFR_creat_) have been shown to perform poorly in African populations, significantly underestimating the overall burden of CKD when compared to gold-standard measured GFR (mGFR) determined by Iohexol plasma clearance.(13-15)”*

We added here some additional supporting references (Bukabau et al, reference 14; Wyatt et al, reference 15) in addition to the existing reference 13 (Fabian et al), which outlines the results of a large multinational study across Malawi, Uganda and South Africa which showed that creatinine-based eGFR equations perform poorly in these populations when compared to gold-standard measured GFR (mGFR):

*Fabian J, Kalyesubula R, Mkandawire J, Hansen CH, Nitsch D, Musenge E, et al. Measurement of kidney function in Malawi, South Africa, and Uganda: a multicentre cohort study. Lancet Glob Health. 2022;10(8):e1159-e69.*

We have also added some additional detail to clarify that the poor performance of creatinine-based eGFR is relative to the performance of estimating equations developed and modelled among predominantly North American or European populations, but used among continental African populations with little validation (Introduction, **pages 5 - 6 lines 70 to 76**).

Some further more specific detail about the degree of inaccuracy of creatinine-based eGFR compared to gold-standard measured GFR (mGFR) specifically in the Malawi cohort of this previous study is outlined on **page 6, lines 76 to 80**:

*“In Malawi, population prevalence of eGFR less than 60ml/min/1.73m^2^ determined using the internationally recommended creatinine-based CKD-EPI (2021) equation(14) is only 0.49% (95% CI 0.31 – 0.73) and using the CKD-EPI 2009 equation(15) without adjustment for African-American ethnicity, 1.25% (95% CI 0.95 – 1.61), compared to 11.91% (95% CI 10.13 – 13.69) when using imputed GFR modelled on Iohexol mGFR.(13)”*

This data shows that serum creatinine-based eGFR significantly underestimates CKD prevalence in African populations when compared to gold standard measured GFR.

Finally, we have added some additional detail on the potential reasons for why serum creatinine may perform less well than cystatin C when compared to measured GFR in continental African populations on **page 6, lines 83 – 92**, as follows:

*“The reasons why serum creatinine performs less well than cystatin C when compared to measured GFR in continental African populations are not yet fully understood but may could potentially relate to differences in lean muscle mass (body mass index, body surface area, height and weights are lower when compared to European ancestry and African American cohorts)(13, 17-20). Possible explanations include nutritional factors (such as low red meat consumption, under-five childhood malnutrition and stunting)(21-23), chronic wasting conditions (such as HIV, tuberculosis and endemic infections) and undiagnosed liver disease, all of which can affect muscle mass and therefore creatinine production(24), as well as genetic determinants of serum creatinine.(25)”*

2. **There's some basic difference between CRTN-based and cystatin-based eGFR equations, and this is not very clearly addressed in the international guidelines. Please notice this difference along your study period.**

Response

Yes you are correct, there are differences between the two equations. Both creatinine-based and cystatin C-based eGFR equations incorporate age, sex and either serum creatinine or serum cystatin C levels. Traditionally creatinine-based eGFR equations also included race-based coefficients however these have been removed from more recently developed and validated equations.

We have added a new supplementary file, **Supplementary file S2**, outlining the full details of all eGFR equations to be used.

Reference to this is now made in the Methods section under ‘eGFR equations’ (**page 19, line 402**):

*“Details of all eGFR equations are provided in full in* ***Supplementary File S2”.***

Please note that as a result of this addition, the names of some other supplementary files have now been updated (the previous supplementary file S2 updated to S3, previous file S3 updated to S4, previous file S4 updated to S5, previous supplementary file S5 updated to S6, and previous supplementary file S6 updated to S7).

3. **Your future success can set a good example to follow because such matters are mostly not attended in large part of Africa**.

Response:

Thank you very much, we greatly appreciate your positive and encouraging feedback. We could not agree more that there is an urgent need to generate high quality data on kidney disease in Africa.

**Reviewer 2**

**The manuscript addresses an important clinical topic, and the focus on eGFR estimation using cystatin C is a valuable approach that may contribute meaningful insights to the field. The topic is timely and relevant, and the study design has potential to generate clinically applicable findings.**

Thank you very much, we greatly appreciate your positive and encouraging feedback.

1. **However, there are several points that would benefit from clarification and alignment. First, the title does not appear to fully reflect the methodology described; revising it to better match the study design may enhance accuracy and clarity.**

Response

Thank you for this feedback. To provide more clarity and better align with the specific objectives, we have revised the title to:

*Risk factors for decline in estimated glomerular filtration rate amongst Malawian adults living in rural Karonga: protocol for a prospective cohort study*

2. **Second, there seems to be some inconsistency between the stated objectives and the methods employed. In particular, while the methodology emphasizes measuring eGFR using cystatin C, the objective is framed as assessing the rate of eGFR decline. It would be helpful to clarify the rationale for this approach or adjust the objectives accordingly.**

Response

Thank you, the Aims and Objectives (**page 8, lines 127 – 131**) states that we plan to assess risk factors for eGFR decline, with eGFR calculated using both serum cystatin C (eGFR_cysC_) and serum creatinine (eGFR_creat_). In order to assess eGFR decline, eGFR_cysC_ and eGFR_creat_ will be calculated at different time points using serum cystatin C and creatinine measured on samples collected at those time points. The eGFR equations that will be used are outlined under Analysis; eGFR equations (now moved to **page 19, lines 388-402**).

Under Methods; Laboratory Methods (**page 16, lines 321-324**) we explained that “Serum samples from this study, and individual level paired serum samples from the HLM-LTC survey, will be tested for serum cystatin C (immunoturbidimetric method, standardised using ERM- DA471/IFCC reference material) and creatinine (kinetic compensated Jaffe method, method A, standardised to an isotope-dilution mass spectroscopy [IDMS] assay).” Under Methods; Participants (**page 10, lines 173-185**) and in Fig 2, we indicated that eligible participants already had to have serum cystatin C tested on a historical stored serum sample to take part, however we have added clarification (lines 183-184) that this baseline sample was collected between 2013 and 2019.

In this way, we aim to have a total of up to three serum samples per participant tested for cystatin and creatinine: a baseline sample (2013-19), an HLM-LTC sample (2021-25) and an Impso study sample (2024-25). We have added clarification to explicitly state this at the start of the Analysis section (Methods; Analysis; eGFR equations, **page 19, lines 389-393**) and in doing so have moved the section ‘eGFR equations’ so that it now comes before the ‘Outcome Measures’ section. We have also added the word “paired” under Methods; Analysis; Outcome Measures (**page 20, line 411**) to clarify that each individual participant will have eGFR calculated from cystatin C and creatinine tested on longitudinal paired samples. We hope this improves the coherence and understandability of the manuscript.

In the section on planned outcome measures (Methods; Analysis; Outcome Measures, **now pages 20-21 lines 414-449**) we outlined the different ways in which we plan to assess both eGFR_cysC_ and eGFR_creat_ decline; this aligns with the overall study objective. The outcome measures for assessing eGFR decline include not just annual rate of eGFR decline (secondary outcome measure 4) but also measures of percentage eGFR decline, change in eGFR category, annual rate of eGFR decline, incidence of CKD and incidence of kidney failure.

3. **Additionally, it is not entirely clear how many times eGFR was measured and at what intervals. Providing explicit details on the number and timing of measurements would improve methodological transparency and reproducibility. Overall, addressing these points would strengthen the coherence and interpretability of the manuscript.**

Response:

Thank you, we have now addressed this in response to your second comment, through the additional explanation added under (Methods; Analysis; eGFR equations, to **page 19, lines 389-393**, which reads as follows:

*“By the end of the study, each participant will have up to three stored serum samples tested for cystatin C and creatinine, allowing calculation of eGFRcysc and eGFRcreat at different points in time: (i) baseline (blood collected between 2013 and 2019); (ii) HLM LTC survey (blood collected between 2021 and 2025); and (iii) Impso study (blood collected between 2024 and 2025). HLM LTC and Impso study samples are collected a minimum of 90 days apart”.*

We hope this improves the clarity of manuscript. It is anticipated that for each participant the time interval between the baseline sample (i) and HLM sample (ii) will be around seven years on average, and interval between the HLM sample (ii) and the Impso study sample (iii) will be a minimum 90 days, but data on the actual time intervals will be presented in the main results paper once the study is complete.

**References**

1. Crampin AC, Dube A, Mboma S, Price A, Chihana M, Jahn A, Baschieri A, Molesworth A, Mwaiyeghele E, Branson K, Floyd S, McGrath N, Fine PE, French N, Glynn JR, Zaba B. Profile: the Karonga Health and Demographic Surveillance System. Int J Epidemiol. 2012 Jun;41(3):676-85. doi: 10.1093/ije/dys088. Epub 2012 Jun 22. PMID: 22729235; PMCID: PMC3396313.
