# Supplementary material for: Risk factors for decline in estimated glomerular filtration rate amongst Malawian adults living in rural Karonga: Protocol for a prospective cohort study using cystatin C- and creatinine-based eGFR
Source: PLoS One. 2026 Jul 27;21(7):e0329042. doi: 10.1371/journal.pone.0329042 (PMC13405090; doi:10.1371/journal.pone.0329042)
Supplement: Attachment — Submitted filename: Response to reviewers_April 2026.docx [file pone.0329042.s012.docx]

**PONE-D-25-34787: Response to reviewers**

*Please note these are the responses to the second round of reviewers. Please see documents resubmitted 31 August 2025 for the responses to the first round of reviewers.*

**Editor comments and journal requirements**

1. **If applicable, we recommend that you deposit your laboratory protocols in** [**protocols.io**](https://url.uk.m.mimecastprotect.com/s/b24kCBPAmUMxLMof6hAS2JhOv?domain=protocols.io) **to enhance the reproducibility of your results.** [**Protocols.io**](https://url.uk.m.mimecastprotect.com/s/33gfCDQDofn78nPtAiNSjXNsp?domain=protocols.io) **assigns your protocol its own identifier (DOI) so that it can be cited independently in the future.**

Response:

Thank you, we have now done this. The following DOI has been reserved (06/04/2025) where the protocol should be available within a few days: <https://dx.doi.org/10.17504/protocols.io.rm7vz4rnxlx1/v1>

**Additional comments to Editor**

1. After responding to Reviewer 3’s comments, the Abstract was 314 words, therefore some additional words have been cut out to ensure compliance with the 300 word limit (see tracked changes version).
2. As a result of the revisions, we have updated the heading titles and page numbers in Supplementary file S7 STROBE checklist and have re-uploaded this in both tracked changes and clean copy formats.

**Reviewer comments**

**Reviewer 1**

**The response and revision from the authors are thorough and intensive. I do not have further comment.**

Response

Thank you very much for the promising feedback on our revisions. Your time in re-reviewing our manuscript is much appreciated.

**Reviewer 3**

**This protocol is recommended for acceptance with minor revisions, as it is scientifically sound, ethically robust, and methodologically advanced. The study addresses an important evidence gap in African chronic kidney disease research through the innovative use of cystatin C–based estimated glomerular filtration rate and long-term longitudinal data from a well-established population cohort. Its rigorous methodological framework, comprehensive ethical considerations, and strong community engagement enhance both internal validity and ethical integrity. Furthermore, the findings are likely to have significant implications for clinical practice, epidemiological surveillance, and health policy in sub-Saharan Africa, underscoring the protocol’s high relevance to global health research priorities.**

Response

Thank you very much for the encouraging feedback and for the time taken to review our manuscript. We have addressed each of your comments in turn, and hope these revisions have strengthened the protocol.

1. **TITLE: The current title is good, but you might consider explicitly reflecting the biomarker innovation. Optional alternative: Risk factors for long-term decline in kidney function among Malawian adults living in rural Karonga: a prospective cohort study using cystatin C–based eGFR**

Response

As per your recommendation we have revised the title to include extra detail on the biomarkers used (both cystatin C and creatinine – see Aims and Objectives; page 8, lines 133 - 137). The updated title reads as follows:

*Risk factors for long-term decline in kidney function among Malawian adults living in rural Karonga: a prospective cohort study using cystatin C- and creatinine-based eGFR*

2. **ABSTRACT (Methods, pages 3 – 4, lines 12 – 30): Kindly state the year the study was carried out, and when the cohort will be accessed subsequently.**

Response

We have added the years for recruitment to the current study, in addition to the years when the two previous linked population-based surveys were conducted, in place of the previous statement “over five years apart” (Abstract, page 3, lines 16 – 16). All study participants are nested within a Health and Demographic Surveillance Site (HDSS), within which follow-up for deaths and migration is continuous (see Methods; Study Design and Setting; pages 8 – 9, lines 149 – 157). To add clarity on this within the Abstract, we have replaced the term “general population cohort” with “Health and Demographic Surveillance Site” (page 3, lines 13 – 14). Follow-up for additional markers of CKD progression beyond those detailed in the manuscript will occur pending successful funding. This is possible due to the unique identifiers assigned to each participant within the wider HDSS which create capacity for long-term linkage in future work. We have clarified this latter point in the Methods, under Study Design and Setting (page 9, lines 156 – 157).

3. **ABSTRACT (Discussion, page 4, line 32): This should not be tagged as a ‘discussion’ rather, it should be an ‘expected outcome’**

Response

We have maintained the subtitle ‘Discussion’ for consistency with the structure of the rest of the manuscript, in line with the journal’s recommended structure, used by the majority of protocol papers it publishes.(1-6)

4. **METHODS (Methods Heading, now page 8, line 139): You made the study design seem to be a retrospective study. Kindly rephrase appropriately to capture what you did in patient recruitment.**

Response

We apologise for the ambiguity here. Although existing data has been used to define eligibility criteria and will be used for some exposure measurements, the currently described Impso study is assessing novel risk factors in addition to all outcome measures through prospective data collection. We have added the following clarifications:

- In the Abstract (page 3, lines 14 – 16) – sentence amended to clarify that participation in the previous studies was an eligibility requirement rather than being a point of recruitment
- In Abstract (page 3, lines 16 – 18) – clarification that the new household level data, blood and urine samples are being collected prospectively.
- In Methods; Participants (page 10, line 186) – changed “recruiting” to “prospectively recruiting”

5. **METHODS (‘Analysis’, now page 19, line 387): Please delete this, I don’t think it’s the right heading**

Response

We have amended this subheading to ‘Analysis Plan’ (page 19, line 387).

6. **METHODS (‘Sample Size’, now page 15, line 369): The sample size calculation should have been written earlier**

Response

Thank you for alerting us to this. We have moved the entirety of the section titled ‘Sample Size’ to appear earlier within the Methods Section of the manuscript (after ‘Study Design and Setting’ and before ‘Population’ – now page 10, lines 177 – 183).

7. **ETHICS, GOVERNANCE AND REGULATIONS (‘Feedback of results and clinical referral, now page 22, line 466): Preliminary results should have been presented and reported**

Response

Apologies for the lack of clarity here. This was referring to the return of individual-level clinical test results to research participants, and not sharing of the overall study findings which will take place later once analysis is complete. Preliminary results are not yet available but are being worked on and will shared as soon as possible. We have amended this sub-heading and paragraph to provide clarification. It now reads (page 22, lines 473 to 480):

***Feedback of individual participant test results and clinical referral***

*Individual urine dipstick results will be communicated to study participants immediately after testing and will be recorded in health passports. Participants with abnormal baseline urine results requiring follow-up urine collection (see ‘Biological Sample Collection’), will receive results of the laboratory urine microscopy at the follow-up visit.*

*Individual baseline eGFR results are communicated to participants at the point of recruitment, accompanied by provision of general CKD lifestyle advice using a standardised information sheet.*

8. **DISCUSSION (Main heading, now page 24, line 522): This should have also included a discussion of the preliminary findings**

Response

Laboratory tests are still being completed and preliminary analysis is ongoing, so findings cannot yet be shared. Preliminary results will be published separately in an additional manuscript.

**References**

1.	Maia CMM, Damasceno K, Seabra LMJ, Chaves G, Dantas L, de Sousa Júnior FC, et al. Efficacy of sanitization protocols in removing parasites in vegetables: A protocol for a systematic review with meta-analysis. PLoS One. 2022;17(5):e0268258.

2.	Mubanga CM, Malabwa C, Machila N, Simuunza MC. Theileria parva in Zambia: A scoping review protocol. PLoS One. 2026;21(2):e0341896.

3.	Gatica-Rojas V, Cartes-Velásquez R, Soto-Poblete A, Lizama LEC. Postural control telerehabilitation with a low-cost virtual reality protocol for children with cerebral palsy: Protocol for a clinical trial. PLoS One. 2023;18(8):e0268163.

4.	Pereira S, Mesquita-Ferrari RA, Salviatto LTC, Bezerra C, Dalapria V, Mello EDS, et al. Photobiomodulation in post menopause genitourinary syndrome-Study protocol for a randomized, double-blind, controlled clinical protocol. PLoS One. 2024;19(12):e0313324.

5.	Martínez-Borba V, Rodríguez-Márquez AE, Garcés-Arilla S, Peris-Baquero Ó, Navarro-Haro MV, Del Corral-Beamonte E, et al. Effectiveness and acceptability of the unified protocol for the transdiagnostic treatment of emotional disorders in people with long COVID-19: Study protocol for a randomized controlled trial. PLoS One. 2026;21(2):e0342908.

6.	McGlinchey D, Ferns A, Martin CW, Murfin A, Killian K, Row J, et al. Protocol for a study on the mental health and well-being of "Forgotten" first responders in Canada. PLoS One. 2026;21(4):e0346019.
