# Supplementary material for: Risk factors for decline in estimated glomerular filtration rate amongst Malawian adults living in rural Karonga: Protocol for a prospective cohort study using cystatin C- and creatinine-based eGFR
Source: PLoS One. 2026 Jul 27;21(7):e0329042. doi: 10.1371/journal.pone.0329042 (PMC13405090; doi:10.1371/journal.pone.0329042)
Supplement: Peer review history

# Peer review: Decision Letter 0

**DOI:** 10.1371/journal.pone.0329042.r001

**Authors:** Keiko Hosohata

12 Aug 2025

PONE-D-25-34787

Risk factors for long-term decline in kidney function amongst Malawian adults living in rural Karonga: protocol for a prospective cohort study

PLOS ONE

Dear Dr. Snead,

Thank you for submitting your manuscript to PLOS ONE. After careful consideration, we feel that it has merit but does not fully meet PLOS ONE’s publication criteria as it currently stands. Therefore, we invite you to submit a revised version of the manuscript that addresses the points raised during the review process.

Please submit your revised manuscript by Sep 26 2025 11:59PM. If you will need more time than this to complete your revisions, please reply to this message or contact the journal office at plosone@plos.org. When you're ready to submit your revision, log on to https://www.editorialmanager.com/pone/ and select the 'Submissions Needing Revision' folder to locate your manuscript file.

Please include the following items when submitting your revised manuscript:

- A rebuttal letter that responds to each point raised by the academic editor and reviewer(s). You should upload this letter as a separate file labeled 'Response to Reviewers'.
- A marked-up copy of your manuscript that highlights changes made to the original version. You should upload this as a separate file labeled 'Revised Manuscript with Track Changes'.
- An unmarked version of your revised paper without tracked changes. You should upload this as a separate file labeled 'Manuscript'.

If you would like to make changes to your financial disclosure, please include your updated statement in your cover letter. Guidelines for resubmitting your figure files are available below the reviewer comments at the end of this letter.

If applicable, we recommend that you deposit your laboratory protocols in protocols.io to enhance the reproducibility of your results. Protocols.io assigns your protocol its own identifier (DOI) so that it can be cited independently in the future. For instructions see: https://journals.plos.org/plosone/s/submission-guidelines#loc-laboratory-protocols. Additionally, PLOS ONE offers an option for publishing peer-reviewed Lab Protocol articles, which describe protocols hosted on protocols.io. Read more information on sharing protocols at https://plos.org/protocols?utm_medium=editorial-email&utm_source=authorletters&utm_campaign=protocols.

We look forward to receiving your revised manuscript.

Kind regards,

Keiko Hosohata, Ph.D.

Academic Editor

PLOS ONE

Journal requirements:

When submitting your revision, we need you to address these additional requirements.

1. Please ensure that your manuscript meets PLOS ONE's style requirements, including those for file naming. The PLOS ONE style templates can be found at

https://journals.plos.org/plosone/s/file?id=wjVg/PLOSOne_formatting_sample_main_body.pdf and

https://journals.plos.org/plosone/s/file?id=ba62/PLOSOne_formatting_sample_title_authors_affiliations.pdf

2. Please include a complete copy of PLOS’ questionnaire on inclusivity in global research in your revised manuscript. Our policy for research in this area aims to improve transparency in the reporting of research performed outside of researchers’ own country or community. The policy applies to researchers who have travelled to a different country to conduct research, research with Indigenous populations or their lands, and research on cultural artefacts. The questionnaire can also be requested at the journal’s discretion for any other submissions, even if these conditions are not met.  Please find more information on the policy and a link to download a blank copy of the questionnaire here: https://journals.plos.org/plosone/s/best-practices-in-research-reporting. Please upload a completed version of your questionnaire as Supporting Information when you resubmit your manuscript.

3. We note that your Data Availability Statement is currently as follows: [All relevant data are within the manuscript and its Supporting Information files.]

Please confirm at this time whether or not your submission contains all raw data required to replicate the results of your study. Authors must share the “minimal data set” for their submission. PLOS defines the minimal data set to consist of the data required to replicate all study findings reported in the article, as well as related metadata and methods (https://journals.plos.org/plosone/s/data-availability#loc-minimal-data-set-definition).

For example, authors should submit the following data:

- The values behind the means, standard deviations and other measures reported;

- The values used to build graphs;

- The points extracted from images for analysis.

Authors do not need to submit their entire data set if only a portion of the data was used in the reported study.

If your submission does not contain these data, please either upload them as Supporting Information files or deposit them to a stable, public repository and provide us with the relevant URLs, DOIs, or accession numbers. For a list of recommended repositories, please see https://journals.plos.org/plosone/s/recommended-repositories

If there are ethical or legal restrictions on sharing a de-identified data set, please explain them in detail (e.g., data contain potentially sensitive information, data are owned by a third-party organization, etc.) and who has imposed them (e.g., an ethics committee). Please also provide contact information for a data access committee, ethics committee, or other institutional body to which data requests may be sent. If data are owned by a third party, please indicate how others may request data access.

4. Your ethics statement should only appear in the Methods section of your manuscript. If your ethics statement is written in any section besides the Methods, please move it to the Methods section and delete it from any other section. Please ensure that your ethics statement is included in your manuscript, as the ethics statement entered into the online submission form will not be published alongside your manuscript.

5. We note that Figure 1 in your submission contain [map/satellite] images which may be copyrighted. All PLOS content is published under the Creative Commons Attribution License (CC BY 4.0), which means that the manuscript, images, and Supporting Information files will be freely available online, and any third party is permitted to access, download, copy, distribute, and use these materials in any way, even commercially, with proper attribution. For these reasons, we cannot publish previously copyrighted maps or satellite images created using proprietary data, such as Google software (Google Maps, Street View, and Earth). For more information, see our copyright guidelines: http://journals.plos.org/plosone/s/licenses-and-copyright.

We require you to either (1) present written permission from the copyright holder to publish these figures specifically under the CC BY 4.0 license, or (2) remove the figures from your submission:

1. You may seek permission from the original copyright holder of Figure 1 to publish the content specifically under the CC BY 4.0 license.

We recommend that you contact the original copyright holder with the Content Permission Form (http://journals.plos.org/plosone/s/file?id=7c09/content-permission-form.pdf) and the following text:

“I request permission for the open-access journal PLOS ONE to publish XXX under the Creative Commons Attribution License (CCAL) CC BY 4.0 (http://creativecommons.org/licenses/by/4.0/). Please be aware that this license allows unrestricted use and distribution, even commercially, by third parties. Please reply and provide explicit written permission to publish XXX under a CC BY license and complete the attached form.”

Please upload the completed Content Permission Form or other proof of granted permissions as an "Other" file with your submission.

In the figure caption of the copyrighted figure, please include the following text: “Reprinted from [ref] under a CC BY license, with permission from [name of publisher], original copyright [original copyright year].”

2. If you are unable to obtain permission from the original copyright holder to publish these figures under the CC BY 4.0 license or if the copyright holder’s requirements are incompatible with the CC BY 4.0 license, please either i) remove the figure or ii) supply a replacement figure that complies with the CC BY 4.0 license. Please check copyright information on all replacement figures and update the figure caption with source information. If applicable, please specify in the figure caption text when a figure is similar but not identical to the original image and is therefore for illustrative purposes only.

The following resources for replacing copyrighted map figures may be helpful

USGS National Map Viewer (public domain): http://viewer.nationalmap.gov/viewer/

The Gateway to Astronaut Photography of Earth (public domain): http://eol.jsc.nasa.gov/sseop/clickmap/

Maps at the CIA (public domain): https://www.cia.gov/library/publications/the-world-factbook/index.html and https://www.cia.gov/library/publications/cia-maps-publications/index.html

NASA Earth Observatory (public domain): http://earthobservatory.nasa.gov/

Landsat: http://landsat.visibleearth.nasa.gov/

USGS EROS (Earth Resources Observatory and Science (EROS) Center) (public domain): http://eros.usgs.gov/#

Natural Earth (public domain): http://www.naturalearthdata.com/

6. If the reviewer comments include a recommendation to cite specific previously published works, please review and evaluate these publications to determine whether they are relevant and should be cited. There is no requirement to cite these works unless the editor has indicated otherwise.

Additional Editor Comments (if provided):

[Note: HTML markup is below. Please do not edit.]

Reviewers' comments:

Reviewer's Responses to Questions

**Comments to the Author**

1. Does the manuscript provide a valid rationale for the proposed study, with clearly identified and justified research questions?

The research question outlined is expected to address a valid academic problem or topic and contribute to the base of knowledge in the field.

Reviewer #1: Yes

Reviewer #2: Partly

**********

2. Is the protocol technically sound and planned in a manner that will lead to a meaningful outcome and allow testing the stated hypotheses?

The manuscript should describe the methods in sufficient detail to prevent undisclosed flexibility in the experimental procedure or analysis pipeline, including sufficient outcome-neutral conditions (e.g. necessary controls, absence of floor or ceiling effects) to test the proposed hypotheses and a statistical power analysis where applicable. As there may be aspects of the methodology and analysis which can only be refined once the work is undertaken, authors should outline potential assumptions and explicitly describe what aspects of the proposed analyses, if any, are exploratory.

Reviewer #1: Yes

Reviewer #2: Partly

**********

3. Is the methodology feasible and described in sufficient detail to allow the work to be replicable?

Descriptions of methods and materials in the protocol should be reported in sufficient detail for another researcher to reproduce all experiments and analyses. The protocol should describe the appropriate controls, sample size calculations, and replication needed to ensure that the data are robust and reproducible.

Reviewer #1: Yes

Reviewer #2: Yes

**********

4. Have the authors described where all data underlying the findings will be made available when the study is complete?

The PLOS Data policy requires authors to make all data underlying the findings described in their manuscript fully available without restriction, with rare exception, at the time of publication. The data should be provided as part of the manuscript or its supporting information, or deposited to a public repository. For example, in addition to summary statistics, the data points behind means, medians and variance measures should be available. If there are restrictions on publicly sharing data—e.g. participant privacy or use of data from a third party—those must be specified.

Reviewer #1: Yes

Reviewer #2: Yes

**********

5. Is the manuscript presented in an intelligible fashion and written in standard English?

PLOS ONE does not copyedit accepted manuscripts, so the language in submitted articles must be clear, correct, and unambiguous. Any typographical or grammatical errors should be corrected at revision, so please note any specific errors here.

Reviewer #1: Yes

Reviewer #2: No

**********

6. Review Comments to the Author

Please use the space provided to explain your answers to the questions above and, if applicable, provide comments about issues authors must address before this protocol can be accepted for publication. You may also include additional comments for the author, including concerns about research or publication ethics.

You may also provide optional suggestions and comments to authors that they might find helpful in planning their study.

(Please upload your review as an attachment if it exceeds 20,000 characters)

Reviewer #1: Authors teamed up with Malawian associates and planned to develop a study system to help clarify the CKD prevalence and risk factors of kidney function decline. This manuscript is descriptive work plan worth encouraging. I had experience to work in that country and understand how much efforts would be necessary to overcome the gap between contemporary medical system and that in Malawi. Comments are as the followings.

1. Readers would like to understand why authors suggested that CRTN-based eGFR is not so suitable in Africans.

2. There's some basic difference between CRTN-based and cystatin-based eGFR equations, and this is not very clearly addressed in the international guidelines. Please notice this difference along your study period.

3. Your future success can set a good example to follow because such matters are mostly not attended in large part of Africa.

Reviewer #2: The manuscript addresses an important clinical topic, and the focus on eGFR estimation using cystatin C is a valuable approach that may contribute meaningful insights to the field. The topic is timely and relevant, and the study design has potential to generate clinically applicable findings.

However, there are several points that would benefit from clarification and alignment. First, the title does not appear to fully reflect the methodology described; revising it to better match the study design may enhance accuracy and clarity. Second, there seems to be some inconsistency between the stated objectives and the methods employed. In particular, while the methodology emphasizes measuring eGFR using cystatin C, the objective is framed as assessing the rate of eGFR decline. It would be helpful to clarify the rationale for this approach or adjust the objectives accordingly.

Additionally, it is not entirely clear how many times eGFR was measured and at what intervals. Providing explicit details on the number and timing of measurements would improve methodological transparency and reproducibility. Overall, addressing these points would strengthen the coherence and interpretability of the manuscript.

**********

7. PLOS authors have the option to publish the peer review history of their article (what does this mean?). If published, this will include your full peer review and any attached files.

If you choose “no”, your identity will remain anonymous but your review may still be made public.

**Do you want your identity to be public for this peer review?**  For information about this choice, including consent withdrawal, please see our Privacy Policy.

Reviewer #1: **Yes:** Jer-Ming Chang

Reviewer #2: No

**********

[NOTE: If reviewer comments were submitted as an attachment file, they will be attached to this email and accessible via the submission site. Please log into your account, locate the manuscript record, and check for the action link "View Attachments". If this link does not appear, there are no attachment files.]

While revising your submission, please upload your figure files to the Preflight Analysis and Conversion Engine (PACE) digital diagnostic tool, https://pacev2.apexcovantage.com/. PACE helps ensure that figures meet PLOS requirements. To use PACE, you must first register as a user. Registration is free. Then, login and navigate to the UPLOAD tab, where you will find detailed instructions on how to use the tool. If you encounter any issues or have any questions when using PACE, please email PLOS at figures@plos.org. Please note that Supporting Information files do not need this step.

# Author comment: Author response to Decision Letter 1

**DOI:** 10.1371/journal.pone.0329042.r002

31 Aug 2025

Editor comments and journal requirements

1. Please ensure that your manuscript meets PLOS ONE's style requirements, including those for file naming. The PLOS ONE style templates can be found at https://journals.plos.org/plosone/s/file?id=wjVg/PLOSOne_formatting_sample_main_body.pdf and https://journals.plos.org/plosone/s/file?id=ba62/PLOSOne_formatting_sample_title_authors_affiliations.pdf

Response:

We have double checked all the submitted documents and can confirm the resubmitted documents meet the PLOS ONE style requirements including those for file naming and formatting.

2. Please include a complete copy of PLOS’ questionnaire on inclusivity in global research in your revised manuscript. Our policy for research in this area aims to improve transparency in the reporting of research performed outside of researchers’ own country or community. The policy applies to researchers who have travelled to a different country to conduct research, research with Indigenous populations or their lands, and research on cultural artefacts. The questionnaire can also be requested at the journal’s discretion for any other submissions, even if these conditions are not met. Please find more information on the policy and a link to download a blank copy of the questionnaire here: https://journals.plos.org/plosone/s/best-practices-in-research-reporting. Please upload a completed version of your questionnaire as Supporting Information when you resubmit your manuscript.

Response:

Thank you very much, we have now completed this documented and uploaded it under Supporting Information.

3. We note that your Data Availability Statement is currently as follows: [All relevant data are within the manuscript and its Supporting Information files.] Please confirm at this time whether or not your submission contains all raw data required to replicate the results of your study. Authors must share the “minimal data set” for their submission. PLOS defines the minimal data set to consist of the data required to replicate all study findings reported in the article, as well as related metadata and methods (https://journals.plos.org/plosone/s/data-availability#loc-minimal-data-set-definition).

For example, authors should submit the following data:

- The values behind the means, standard deviations and other measures reported;

- The values used to build graphs;

- The points extracted from images for analysis.

Authors do not need to submit their entire data set if only a portion of the data was used in the reported study.

If your submission does not contain these data, please either upload them as Supporting Information files or deposit them to a stable, public repository and provide us with the relevant URLs, DOIs, or accession numbers. For a list of recommended repositories, please see https://journals.plos.org/plosone/s/recommended-repositories

If there are ethical or legal restrictions on sharing a de-identified data set, please explain them in detail (e.g., data contain potentially sensitive information, data are owned by a third-party organization, etc.) and who has imposed them (e.g., an ethics committee). Please also provide contact information for a data access committee, ethics committee, or other institutional body to which data requests may be sent. If data are owned by a third party, please indicate how others may request data access.

Response:

Only a portion of the data from the MEIRU NCD survey and the African Research on Kidney Disease (ARK) Consortium study was used to create the tables in Supplementary files S4 and S5 submitted with this protocol paper (these files were previously named S3 and S4 but have been updated to S4 and S5 due to addition of new supplementary file S2 in response to reviewer 1 comments). No pilot data or outcome data from the study is reported within the main article.

We have not submitted the full datasets but have revised our Data Availability Statement and provide clarity on how readers can access the raw data.

Data from both these studies is publicly available. The full data from the MEIRU NCD survey can be accessed via the following public repository; https://datacompass.lshtm.ac.uk/id/eprint/3834/; with some creatinine data available in an additional public repository; https://datacompass.lshtm.ac.uk/id/eprint/1150/

Data from the ARK study is available publicly at: https://github.com/ARKconsortium/iohexol_mGFR_eGFR.

This is a standardised set of relevant data from all three study sites involved in that study: Malawi, South Africa and Uganda. The dataset includes deidentified individual participant data related to estimated and measured GFR, with accompanying analytic code used for development of a new creatinine-based GFR-estimating equation.

The specific merged dataset used to create supplementary tables S4 and S5 used anonymised participant-level data from both the MEIRU NCD survey and the Malawi arm of the ARK study (all individuals had taken part in the MEIRU NCD survey; a subset had additional taken part in the ARK study). This data will be publicly available by the time the currently described study is completed and published, but is not yet accessible in a public repository as work is ongoing to finalise open datasets. However while this work is ongoing the dataset can be accessed on request through application to the MEIRU data access committee (mia.crampin@glasgow.ac.uk; paul.kambiya@meiru.mw). Deidentified datasets from the currently ongoing Impso study, described in this protocol paper, will also be made publicly available once analysis is complete.

4. Your ethics statement should only appear in the Methods section of your manuscript. If your ethics statement is written in any section besides the Methods, please move it to the Methods section and delete it from any other section. Please ensure that your ethics statement is included in your manuscript, as the ethics statement entered into the online submission form will not be published alongside your manuscript.

Response:

We have doubled checked and confirm that the ethics statement only appeared in the Methods section of our Manuscript, under the Methods subsection, “Ethics, Governance and Regulations; Ethical approval” – please see page 21-22, lines 468-486. We have amended the subheading from “ethical approval” to “ethical approval and informed consent” and added an additional statement on informed consent. The specific details of the informed consent process are outlined in the earlier Methods subsection, “Recruitment and consent” (pages 11-12, lines 211 - 226).

5. We note that Figure 1 in your submission contain [map/satellite] images which may be copyrighted. All PLOS content is published under the Creative Commons Attribution License (CC BY 4.0), which means that the manuscript, images, and Supporting Information files will be freely available online, and any third party is permitted to access, download, copy, distribute, and use these materials in any way, even commercially, with proper attribution. For these reasons, we cannot publish previously copyrighted maps or satellite images created using proprietary data, such as Google software (Google Maps, Street View, and Earth). For more information, see our copyright guidelines: http://journals.plos.org/plosone/s/licenses-and-copyright.

We require you to either (1) present written permission from the copyright holder to publish these figures specifically under the CC BY 4.0 license, or (2) remove the figures from your submission:

1. You may seek permission from the original copyright holder of Figure 1 to publish the content specifically under the CC BY 4.0 license.

We recommend that you contact the original copyright holder with the Content Permission Form (http://journals.plos.org/plosone/s/file?id=7c09/content-permission-form.pdf) and the following text:

“I request permission for the open-access journal PLOS ONE to publish XXX under the Creative Commons Attribution License (CCAL) CC BY 4.0 (http://creativecommons.org/licenses/by/4.0/). Please be aware that this license allows unrestricted use and distribution, even commercially, by third parties. Please reply and provide explicit written permission to publish XXX under a CC BY license and complete the attached form.”

Please upload the completed Content Permission Form or other proof of granted permissions as an "Other" file with your submission.

In the figure caption of the copyrighted figure, please include the following text: “Reprinted from [ref] under a CC BY license, with permission from [name of publisher], original copyright [original copyright year].”

2. If you are unable to obtain permission from the original copyright holder to publish these figures under the CC BY 4.0 license or if the copyright holder’s requirements are incompatible with the CC BY 4.0 license, please either i) remove the figure or ii) supply a replacement figure that complies with the CC BY 4.0 license. Please check copyright information on all replacement figures and update the figure caption with source information. If applicable, please specify in the figure caption text when a figure is similar but not identical to the original image and is therefore for illustrative purposes only.

The following resources for replacing copyrighted map figures may be helpful

USGS National Map Viewer (public domain): http://viewer.nationalmap.gov/viewer/

The Gateway to Astronaut Photography of Earth (public domain): http://eol.jsc.nasa.gov/sseop/clickmap/

Maps at the CIA (public domain): https://www.cia.gov/library/publications/the-world-factbook/index.html and https://www.cia.gov/library/publications/cia-maps-publications/index.html

NASA Earth Observatory (public domain): http://earthobservatory.nasa.gov/

Landsat: http://landsat.visibleearth.nasa.gov/

USGS EROS (Earth Resources Observatory and Science (EROS) Center) (public domain): http://eros.usgs.gov/#

Natural Earth (public domain): http://www.naturalearthdata.com/

Response:

We have amended the image in Figure 1 to just include just the zoomed in map of the Karonga Health and Demographic Surveillance Site. One of the authors of this manuscript, AD, generated this map presented in Figure 1 using Quantum Geographic Information System (QGIS) a free and open-source software that allows users to create, edit, visualize, analyse, and publish geospatial information. From 2002 to 2005, GPS units were used by project officers and village motivators to track village and cluster boundaries.(1) Since 2005 to date more cluster boundary data has been collected and stored by the project. We loaded this project data into QGIS 3.32 Lima in order to generate the map. The QGIS user guidance states that:

“There is no requirement to mention QGIS for maps produced with it. It is of course really welcome if you would like to add a note saying that the map was produced with QGIS. “Made with QGIS” or “Map created using the Free and Open Source QGIS” are good examples of such a note. Do not say ©QGIS as QGIS does not hold copyrights on your work”.

This guidance can be found here:

https://qgis.org/resources/support/faq/

We have therefore amended the caption of Figure 1 to include the QGIS-endorsed statement:

Map created using the Free and Open Source Quantum Geographic Information System (QGIS): https://qgis.org/

Since QGIS state they do not hold copyrights on images created using their software, we could not ask them to complete the Content Permission Form, however please do let us know if we need to provide any further information or clarification with regards to this image.

6. If the reviewer comments include a recommendation to cite specific previously published works, please review and evaluate these publications to determine whether they are relevant and should be cited. There is no requirement to cite these works unless the editor has indicated otherwise.

Response:

Not applicable; the reviewer comments did not include any recommendations to cite specific works.

Reviewer comments

Reviewer 1

Authors teamed up with Malawian associates and planned to develop a study system to help clarify the CKD prevalence and risk factors of kidney function decline. This manuscript is descriptive work plan worth encouraging. I had experience to work in that country and understand how much efforts would be necessary to overcome the gap between contemporary medical system and that in Malawi. Comments are as the followings.

Thank you very much for the positive and encouraging feedback. All co-authors, Malawian and international, are full team members of the study, which aims to investigate risk factors for kidney function decline (see Introduction; Aims and Objectives, page 8 lines 127-131).

1. Readers would like to understand why authors suggested that CRTN-based eGFR is not so suitable in Africans.

Response

We have outlined the background as to why creatinine-based eGFR performs poorly compared to cystatin C and gold standard measured GFR in the Please see Introduction (page 5, lines 67 to 70) which outlines the background regarding the difference in performance between creatinine-based eGFR and gold standard measured GFR in African populations:

“Importantly, the most readily available creatinine-based equations for estimating glomerular filtration rate (eGFRcreat) have been shown to perform poorly in African populations, significantly underestimating the overall burden of CKD when compared to gold-standard measured GFR (mGFR) determined by Iohexol plasma clearance.(13-15)”

We added here some additional supporting references (Bukabau et al, reference 14; Wyatt et al, reference 15) in addition to the existing reference 13 (Fabian et al), which outlines the results of a large multinational study across Malawi, Uganda and South Africa which showed that creatinine-based eGFR equations perform poorly in these populations when compared to gold-standard measured GFR (mGFR):

Fabian J, Kalyesubula R, Mkandawire J, Hansen CH, Nitsch D, Musenge E, et al. Measurement of kidney function in Malawi, South Africa, and Uganda: a multicentre cohort study. Lancet Glob Health. 2022;10(8):e1159-e69.

We have also added some additional detail to clarify that the poor performance of creatinine-based eGFR is relative to the performance of estimating equations developed and modelled among predominantly North American or European populations, but used among continental African populations with little validation (Introduction, pages 5 - 6 lines 70 to 76).

Some further more specific detail about the degree of inaccuracy of creatinine-based eGFR compared to gold-standard measured GFR (mGFR) specifically in the Malawi cohort of this previous study is outlined on page 6, lines 76 to 80:

“In Malawi, population prevalence of eGFR less than 60ml/min/1.73m2 determined using the internationally recommended creatinine-based CKD-EPI (2021) equation(14) is only 0.49% (95% CI 0.31 – 0.73) and using the CKD-EPI 2009 equation(15) without adjustment for African-American ethnicity, 1.25% (95% CI 0.95 – 1.61), compared to 11.91% (95% CI 10.13 – 13.69) when using imputed GFR modelled on Iohexol mGFR.(13)”

This data shows that serum creatinine-based eGFR significantly underestimates CKD prevalence in African populations when compared to gold standard measured GFR.

Finally, we have added some additional detail on the potential reasons for why serum creatinine may perform less well than cystatin C when compared to measured GFR in continental African populations on page 6, lines 83 – 92, as follows:

“The reasons why serum creatinine performs less well than cystatin C when compared to measured GFR in continental African populations are not yet fully understood but may could potentially relate to differences in lean muscle mass (body mass index, body surface area, height and weights are lower when compared to European ancestry and African American cohorts)(13, 17-20). Possible explanations include nutritional factors (such as low red meat consumption, under-five childhood malnutrition and stunting)(21-23), chronic wasting conditions (such as HIV, tuberculosis and endemic infections) and undiagnosed liver disease, all of which can affect muscle mass and therefore creatinine production(24), as well as genetic determinants

Attachment Submitted filename: Response to reviewers.docx

# Peer review: Decision Letter 1

**DOI:** 10.1371/journal.pone.0329042.r003

**Authors:** Johanna Pruller

29 Mar 2026

PONE-D-25-34787R1

Risk factors for decline in estimated glomerular filtration rate amongst Malawian adults living in rural Karonga: protocol for a prospective cohort study

PLOS One

Dear Dr. Snead,

Thank you for submitting your manuscript to PLOS ONE. After careful consideration, we feel that it has merit but does not fully meet PLOS ONE’s publication criteria as it currently stands. Therefore, we invite you to submit a revised version of the manuscript that addresses the points raised during the review process.

==============================

The manuscript has been evaluated by one reviewer and their comments are available below.

The reviewer has raised a few concerns that need attention. Could you please revise the manuscript to carefully address the concerns raised?

==============================

Please submit your revised manuscript by May 13 2026 11:59PM. If you will need more time than this to complete your revisions, please reply to this message or contact the journal office at plosone@plos.org. When you're ready to submit your revision, log on to https://www.editorialmanager.com/pone/ and select the 'Submissions Needing Revision' folder to locate your manuscript file.

Please include the following items when submitting your revised manuscript:

- A letter that responds to each point raised by the academic editor and reviewer(s). You should upload this letter as a separate file labeled 'Response to Reviewers'.
- A marked-up copy of your manuscript that highlights changes made to the original version. You should upload this as a separate file labeled 'Revised Manuscript with Track Changes'.
- An unmarked version of your revised paper without tracked changes. You should upload this as a separate file labeled 'Manuscript'.

If you would like to make changes to your financial disclosure, please include your updated statement in your cover letter. Guidelines for resubmitting your figure files are available below the reviewer comments at the end of this letter.

If applicable, we recommend that you deposit your laboratory protocols in protocols.io to enhance the reproducibility of your results. Protocols.io assigns your protocol its own identifier (DOI) so that it can be cited independently in the future. For instructions see: https://journals.plos.org/plosone/s/submission-guidelines#loc-laboratory-protocols. Additionally, PLOS ONE offers an option for publishing peer-reviewed Lab Protocol articles, which describe protocols hosted on protocols.io. Read more information on sharing protocols at https://plos.org/protocols?utm_medium=editorial-email&utm_source=authorletters&utm_campaign=protocols.

As the corresponding author, your ORCID iD is verified in the submission system and will appear in the published article. PLOS supports the use of ORCID, and we encourage all coauthors to register for an ORCID iD and use it as well. Please encourage your coauthors to verify their ORCID iD within the submission system before final acceptance, as unverified ORCID iDs will not appear in the published article. *Only*  the individual author can complete the verification step; PLOS staff *cannot*  verify ORCID iDs on behalf of authors.

We look forward to receiving your revised manuscript.

Kind regards,

Johanna Pruller, Ph.D.

Senior Editor

PLOS One

**Journal Requirements:**

If the reviewer comments include a recommendation to cite specific previously published works, please review and evaluate these publications to determine whether they are relevant and should be cited. There is no requirement to cite these works unless the editor has indicated otherwise.

[Note: HTML markup is below. Please do not edit.]

Reviewers' comments:

Reviewer's Responses to Questions

**Comments to the Author**

1. Does the manuscript provide a valid rationale for the proposed study, with clearly identified and justified research questions?

The research question outlined is expected to address a valid academic problem or topic and contribute to the base of knowledge in the field.

Reviewer #1: Yes

Reviewer #3: Yes

**********

2. Is the protocol technically sound and planned in a manner that will lead to a meaningful outcome and allow testing the stated hypotheses?

The manuscript should describe the methods in sufficient detail to prevent undisclosed flexibility in the experimental procedure or analysis pipeline, including sufficient outcome-neutral conditions (e.g. necessary controls, absence of floor or ceiling effects) to test the proposed hypotheses and a statistical power analysis where applicable. As there may be aspects of the methodology and analysis which can only be refined once the work is undertaken, authors should outline potential assumptions and explicitly describe what aspects of the proposed analyses, if any, are exploratory.

Reviewer #1: Yes

Reviewer #3: Partly

**********

3. Is the methodology feasible and described in sufficient detail to allow the work to be replicable?

Descriptions of methods and materials in the protocol should be reported in sufficient detail for another researcher to reproduce all experiments and analyses. The protocol should describe the appropriate controls, sample size calculations, and replication needed to ensure that the data are robust and reproducible.

Reviewer #1: Yes

Reviewer #3: Yes

**********

4. Have the authors described where all data underlying the findings will be made available when the study is complete?

The PLOS Data policy requires authors to make all data underlying the findings described in their manuscript fully available without restriction, with rare exception, at the time of publication. The data should be provided as part of the manuscript or its supporting information, or deposited to a public repository. For example, in addition to summary statistics, the data points behind means, medians and variance measures should be available. If there are restrictions on publicly sharing data—e.g. participant privacy or use of data from a third party—those must be specified.

Reviewer #1: Yes

Reviewer #3: Yes

**********

5. Is the manuscript presented in an intelligible fashion and written in standard English?

PLOS ONE does not copyedit accepted manuscripts, so the language in submitted articles must be clear, correct, and unambiguous. Any typographical or grammatical errors should be corrected at revision, so please note any specific errors here.

Reviewer #1: Yes

Reviewer #3: Yes

**********

6. Review Comments to the Author

Please use the space provided to explain your answers to the questions above and, if applicable, provide comments about issues authors must address before this protocol can be accepted for publication. You may also include additional comments for the author, including concerns about research or publication ethics.

You may also provide optional suggestions and comments to authors that they might find helpful in planning their study.

(Please upload your review as an attachment if it exceeds 20,000 characters)

Reviewer #1: The response and revision from the authors are thorough and intensive. I do not have further comment.

Reviewer #3: This protocol is recommended for acceptance with minor revisions, as it is scientifically sound, ethically robust, and methodologically advanced. The study addresses an important evidence gap in African chronic kidney disease research through the innovative use of cystatin C–based estimated glomerular filtration rate and long-term longitudinal data from a well-established population cohort. Its rigorous methodological framework, comprehensive ethical considerations, and strong community engagement enhance both internal validity and ethical integrity. Furthermore, the findings are likely to have significant implications for clinical practice, epidemiological surveillance, and health policy in sub-Saharan Africa, underscoring the protocol’s high relevance to global health research priorities.

**********

7. PLOS authors have the option to publish the peer review history of their article (what does this mean?). If published, this will include your full peer review and any attached files.

If you choose “no”, your identity will remain anonymous but your review may still be made public.

**Do you want your identity to be public for this peer review?**  For information about this choice, including consent withdrawal, please see our Privacy Policy.

Reviewer #1: **Yes:** Jer-Ming Chang

Reviewer #3: **Yes:** DR EREKU TORITSEJU OMAGBEMI

**********

[NOTE: If reviewer comments were submitted as an attachment file, they will be attached to this email and accessible via the submission site. Please log into your account, locate the manuscript record, and check for the action link "View Attachments". If this link does not appear, there are no attachment files.]

To ensure your figures meet our technical requirements, please review our figure guidelines: https://journals.plos.org/plosone/s/figures

You may also use PLOS’s free figure tool, NAAS, to help you prepare publication quality figures: https://journals.plos.org/plosone/s/figures#loc-tools-for-figure-preparation.

NAAS will assess whether your figures meet our technical requirements by comparing each figure against our figure specifications.

Attachment Submitted filename: Snead et al_Main manuscript_review.docx

# Author comment: Author response to Decision Letter 2

**DOI:** 10.1371/journal.pone.0329042.r004

7 Apr 2026

Editor comments and journal requirements

1. If applicable, we recommend that you deposit your laboratory protocols in protocols.io to enhance the reproducibility of your results. Protocols.io assigns your protocol its own identifier (DOI) so that it can be cited independently in the future.

Response:

Thank you, we have now done this. The following DOI has been reserved (06/04/2025) where the protocol should be available within a few days: https://dx.doi.org/10.17504/protocols.io.rm7vz4rnxlx1/v1

Additional comments to Editor

1. After responding to Reviewer 3’s comments, the Abstract was 314 words, therefore some additional words have been cut out to ensure compliance with the 300 word limit (see tracked changes version).

2. As a result of the revisions, we have updated the heading titles and page numbers in Supplementary file S7 STROBE checklist and have re-uploaded this in both tracked changes and clean copy formats.

Reviewer comments

Reviewer 1

The response and revision from the authors are thorough and intensive. I do not have further comment.

Response

Thank you very much for the promising feedback on our revisions. Your time in re-reviewing our manuscript is much appreciated.

Reviewer 3

This protocol is recommended for acceptance with minor revisions, as it is scientifically sound, ethically robust, and methodologically advanced. The study addresses an important evidence gap in African chronic kidney disease research through the innovative use of cystatin C–based estimated glomerular filtration rate and long-term longitudinal data from a well-established population cohort. Its rigorous methodological framework, comprehensive ethical considerations, and strong community engagement enhance both internal validity and ethical integrity. Furthermore, the findings are likely to have significant implications for clinical practice, epidemiological surveillance, and health policy in sub-Saharan Africa, underscoring the protocol’s high relevance to global health research priorities.

Response

Thank you very much for the encouraging feedback and for the time taken to review our manuscript. We have addressed each of your comments in turn, and hope these revisions have strengthened the protocol.

1. TITLE: The current title is good, but you might consider explicitly reflecting the biomarker innovation. Optional alternative: Risk factors for long-term decline in kidney function among Malawian adults living in rural Karonga: a prospective cohort study using cystatin C–based eGFR

Response

As per your recommendation we have revised the title to include extra detail on the biomarkers used (both cystatin C and creatinine – see Aims and Objectives; page 8, lines 133 - 137). The updated title reads as follows:

Risk factors for long-term decline in kidney function among Malawian adults living in rural Karonga: a prospective cohort study using cystatin C- and creatinine-based eGFR

2. ABSTRACT (Methods, pages 3 – 4, lines 12 – 30): Kindly state the year the study was carried out, and when the cohort will be accessed subsequently.

Response

We have added the years for recruitment to the current study, in addition to the years when the two previous linked population-based surveys were conducted, in place of the previous statement “over five years apart” (Abstract, page 3, lines 16 – 16). All study participants are nested within a Health and Demographic Surveillance Site (HDSS), within which follow-up for deaths and migration is continuous (see Methods; Study Design and Setting; pages 8 – 9, lines 149 – 157). To add clarity on this within the Abstract, we have replaced the term “general population cohort” with “Health and Demographic Surveillance Site” (page 3, lines 13 – 14). Follow-up for additional markers of CKD progression beyond those detailed in the manuscript will occur pending successful funding. This is possible due to the unique identifiers assigned to each participant within the wider HDSS which create capacity for long-term linkage in future work. We have clarified this latter point in the Methods, under Study Design and Setting (page 9, lines 156 – 157).

3. ABSTRACT (Discussion, page 4, line 32): This should not be tagged as a ‘discussion’ rather, it should be an ‘expected outcome’

Response

We have maintained the subtitle ‘Discussion’ for consistency with the structure of the rest of the manuscript, in line with the journal’s recommended structure, used by the majority of protocol papers it publishes.(1-6)

4. METHODS (Methods Heading, now page 8, line 139): You made the study design seem to be a retrospective study. Kindly rephrase appropriately to capture what you did in patient recruitment.

Response

We apologise for the ambiguity here. Although existing data has been used to define eligibility criteria and will be used for some exposure measurements, the currently described Impso study is assessing novel risk factors in addition to all outcome measures through prospective data collection. We have added the following clarifications:

- In the Abstract (page 3, lines 14 – 16) – sentence amended to clarify that participation in the previous studies was an eligibility requirement rather than being a point of recruitment

- In Abstract (page 3, lines 16 – 18) – clarification that the new household level data, blood and urine samples are being collected prospectively.

- In Methods; Participants (page 10, line 186) – changed “recruiting” to “prospectively recruiting”

5. METHODS (‘Analysis’, now page 19, line 387): Please delete this, I don’t think it’s the right heading

Response

We have amended this subheading to ‘Analysis Plan’ (page 19, line 387).

6. METHODS (‘Sample Size’, now page 15, line 369): The sample size calculation should have been written earlier

Response

Thank you for alerting us to this. We have moved the entirety of the section titled ‘Sample Size’ to appear earlier within the Methods Section of the manuscript (after ‘Study Design and Setting’ and before ‘Population’ – now page 10, lines 177 – 183).

7. ETHICS, GOVERNANCE AND REGULATIONS (‘Feedback of results and clinical referral, now page 22, line 466): Preliminary results should have been presented and reported

Response

Apologies for the lack of clarity here. This was referring to the return of individual-level clinical test results to research participants, and not sharing of the overall study findings which will take place later once analysis is complete. Preliminary results are not yet available but are being worked on and will shared as soon as possible. We have amended this sub-heading and paragraph to provide clarification. It now reads (page 22, lines 473 to 480):

Feedback of individual participant test results and clinical referral

Individual urine dipstick results will be communicated to study participants immediately after testing and will be recorded in health passports. Participants with abnormal baseline urine results requiring follow-up urine collection (see ‘Biological Sample Collection’), will receive results of the laboratory urine microscopy at the follow-up visit.

Individual baseline eGFR results are communicated to participants at the point of recruitment, accompanied by provision of general CKD lifestyle advice using a standardised information sheet.

8. DISCUSSION (Main heading, now page 24, line 522): This should have also included a discussion of the preliminary findings

Response

Laboratory tests are still being completed and preliminary analysis is ongoing, so findings cannot yet be shared. Preliminary results will be published separately in an additional manuscript.

References

1. Maia CMM, Damasceno K, Seabra LMJ, Chaves G, Dantas L, de Sousa Júnior FC, et al. Efficacy of sanitization protocols in removing parasites in vegetables: A protocol for a systematic review with meta-analysis. PLoS One. 2022;17(5):e0268258.

2. Mubanga CM, Malabwa C, Machila N, Simuunza MC. Theileria parva in Zambia: A scoping review protocol. PLoS One. 2026;21(2):e0341896.

3. Gatica-Rojas V, Cartes-Velásquez R, Soto-Poblete A, Lizama LEC. Postural control telerehabilitation with a low-cost virtual reality protocol for children with cerebral palsy: Protocol for a clinical trial. PLoS One. 2023;18(8):e0268163.

4. Pereira S, Mesquita-Ferrari RA, Salviatto LTC, Bezerra C, Dalapria V, Mello EDS, et al. Photobiomodulation in post menopause genitourinary syndrome-Study protocol for a randomized, double-blind, controlled clinical protocol. PLoS One. 2024;19(12):e0313324.

5. Martínez-Borba V, Rodríguez-Márquez AE, Garcés-Arilla S, Peris-Baquero Ó, Navarro-Haro MV, Del Corral-Beamonte E, et al. Effectiveness and acceptability of the unified protocol for the transdiagnostic treatment of emotional disorders in people with long COVID-19: Study protocol for a randomized controlled trial. PLoS One. 2026;21(2):e0342908.

6. McGlinchey D, Ferns A, Martin CW, Murfin A, Killian K, Row J, et al. Protocol for a study on the mental health and well-being of "Forgotten" first responders in Canada. PLoS One. 2026;21(4):e0346019.

Attachment Submitted filename: Response to reviewers_April 2026.docx

# Peer review: Decision Letter 2

**DOI:** 10.1371/journal.pone.0329042.r005

**Authors:** Diego Moriconi

8 Jun 2026

Risk factors for decline in estimated glomerular filtration rate amongst Malawian adults living in rural Karonga: protocol for a prospective cohort study using cystatin C- and creatinine-based eGFR

PONE-D-25-34787R2

Dear Dr. Charlotte May Snead,

We’re pleased to inform you that your manuscript has been judged scientifically suitable for publication and will be formally accepted for publication once it meets all outstanding technical requirements.

Within one week, you’ll receive an e-mail detailing the required amendments. When these have been addressed, you’ll receive a formal acceptance letter and your manuscript will be scheduled for publication.

An invoice will be generated when your article is formally accepted. Please note, if your institution has a publishing partnership with PLOS and your article meets the relevant criteria, all or part of your publication costs will be covered. Please make sure your user information is up-to-date by logging into Editorial Manager at Editorial Manager® and clicking the ‘Update My Information' link at the top of the page. For questions related to billing, please contact billing support.

If your institution or institutions have a press office, please notify them about your upcoming paper to help maximize its impact. If they’ll be preparing press materials, please inform our press team as soon as possible -- no later than 48 hours after receiving the formal acceptance. Your manuscript will remain under strict press embargo until 2 pm Eastern Time on the date of publication. For more information, please contact onepress@plos.org.

Kind regards,

Diego Moriconi

Academic Editor

PLOS One

Additional Editor Comments (optional):

The revised manuscript adequately addresses the reviewers’ comments. Although the protocol remains subject to limitations typical of observational cohort studies, including potential residual confounding and participant attrition over time, these issues are recognised and appropriately discussed by the authors. Overall, the study is scientifically sound and suitable for publication. I recommend acceptance.

Reviewers' comments:

Reviewer's Responses to Questions

**Comments to the Author**

1. Does the manuscript provide a valid rationale for the proposed study, with clearly identified and justified research questions?

The research question outlined is expected to address a valid academic problem or topic and contribute to the base of knowledge in the field.

Reviewer #1: Yes

**********

2. Is the protocol technically sound and planned in a manner that will lead to a meaningful outcome and allow testing the stated hypotheses?

The manuscript should describe the methods in sufficient detail to prevent undisclosed flexibility in the experimental procedure or analysis pipeline, including sufficient outcome-neutral conditions (e.g. necessary controls, absence of floor or ceiling effects) to test the proposed hypotheses and a statistical power analysis where applicable. As there may be aspects of the methodology and analysis which can only be refined once the work is undertaken, authors should outline potential assumptions and explicitly describe what aspects of the proposed analyses, if any, are exploratory.

Reviewer #1: Yes

**********

3. Is the methodology feasible and described in sufficient detail to allow the work to be replicable?

Descriptions of methods and materials in the protocol should be reported in sufficient detail for another researcher to reproduce all experiments and analyses. The protocol should describe the appropriate controls, sample size calculations, and replication needed to ensure that the data are robust and reproducible.

Reviewer #1: Yes

**********

4. Have the authors described where all data underlying the findings will be made available when the study is complete?

The PLOS Data policy requires authors to make all data underlying the findings described in their manuscript fully available without restriction, with rare exception, at the time of publication. The data should be provided as part of the manuscript or its supporting information, or deposited to a public repository. For example, in addition to summary statistics, the data points behind means, medians and variance measures should be available. If there are restrictions on publicly sharing data—e.g. participant privacy or use of data from a third party—those must be specified.

Reviewer #1: Yes

**********

5. Is the manuscript presented in an intelligible fashion and written in standard English?

PLOS ONE does not copyedit accepted manuscripts, so the language in submitted articles must be clear, correct, and unambiguous. Any typographical or grammatical errors should be corrected at revision, so please note any specific errors here.

Reviewer #1: Yes

**********

6. Review Comments to the Author

Please use the space provided to explain your answers to the questions above and, if applicable, provide comments about issues authors must address before this protocol can be accepted for publication. You may also include additional comments for the author, including concerns about research or publication ethics.

You may also provide optional suggestions and comments to authors that they might find helpful in planning their study.

(Please upload your review as an attachment if it exceeds 20,000 characters)

Reviewer #1: Authors have revised the manuscript (again) according to the previous opinions. I do not have further comment.

**********

7. PLOS authors have the option to publish the peer review history of their article (what does this mean?). If published, this will include your full peer review and any attached files.

If you choose “no”, your identity will remain anonymous but your review may still be made public.

**Do you want your identity to be public for this peer review?**  For information about this choice, including consent withdrawal, please see our Privacy Policy.

Reviewer #1: **Yes:** Jer-Ming Chang

**********

# Editor report: Acceptance letter

**DOI:** 10.1371/journal.pone.0329042.r006

**Authors:** Diego Moriconi

PONE-D-25-34787R2

PLOS One

Dear Dr. Snead,

I'm pleased to inform you that your manuscript has been deemed suitable for publication in PLOS One. Congratulations! Your manuscript is now being handed over to our production team.

At this stage, our production department will prepare your paper for publication. This includes ensuring the following:

* All references, tables, and figures are properly cited

* All relevant supporting information is included in the manuscript submission,

* There are no issues that prevent the paper from being properly typeset

You will receive further instructions from the production team, including instructions on how to review your proof when it is ready. Please keep in mind that we are working through a large volume of accepted articles, so please give us a few days to review your paper and let you know the next and final steps.

Lastly, if your institution or institutions have a press office, please let them know about your upcoming paper now to help maximize its impact. If they'll be preparing press materials, please inform our press team within the next 48 hours. Your manuscript will remain under strict press embargo until 2 pm Eastern Time on the date of publication. For more information, please contact onepress@plos.org.

You will receive an invoice from PLOS for your publication fee after your manuscript has reached the completed accept phase. If you receive an email requesting payment before acceptance or for any other service, this may be a phishing scheme. Learn how to identify phishing emails and protect your accounts at https://explore.plos.org/phishing.

If we can help with anything else, please email us at customercare@plos.org.

Thank you for submitting your work to PLOS ONE and supporting open access.

Kind regards,

PLOS ONE Editorial Office Staff

on behalf of

Dr. Diego Moriconi

Academic Editor

PLOS One
